# The combined formulation of brassinolide and pyraclostrobin increases biomass and seed yield by improving photosynthetic capacity in *Arabidopsis thaliana*

**DOI:** 10.3389/fpls.2023.1138563

**Published:** 2023-03-29

**Authors:** Ya-Qi An, Zi-Ting Qin, Dan-Dan Li, Rui-Qi Zhao, Bo-Shi Bi, Da-Wei Wang, De-Jun Ma, Zhen Xi

**Affiliations:** State Key Laboratory of Elemento-Organic Chemistry, and Department of Chemical Biology, National Pesticide Engineering Research Center, Collaborative Innovation Center of Chemical Science and Engineering, College of Chemistry, Nankai University, Tianjin, China

**Keywords:** brassinosteroids, pyraclostrobin, photosynthesis, transcriptomics, metabolomics, yield, *Arabidopsis thaliana*

## Abstract

In the context of global food crisis, applying the phytohormone-brassinosteroids (BRs) in combination with the fungicide-pyraclostrobin (Pyr) was beneficial for plant quality and productivity in several field trials. However, in addition to the benefits of disease control due to the innate fungicidal activity of Pyr, it remains to be understood whether the coapplication of BL+ Pyr exerts additional growth-promoting effects. For this purpose, the effects of BL treatment, Pyr treatment, and BL+ Pyr treatment in *Arabidopsis thaliana* were compared. The results showed that the yield increased at a rate of 25.6% in the BL+Pyr group and 9.7% in the BL group, but no significant change was observed in the Pyr group. Furthermore, the BL+Pyr treatment increased the fresh weight of both the leaves and the inflorescences. In contrast, the Pyr and BL treatments only increased the fresh weight of leaves and inflorescences, respectively. Additionally, the BL + Pyr treatment increased the P_n_, G_s_, T_r_, V_c, max_, J_max_, V_TPU_, ETR, F_v_’/F_m_’, ΦPSII, Rd, AYE and Rubisco enzyme activity by 26%, 38%, 40%, 16%, 19%, 15%, 9%, 10%, 17%, 179%, 18% and 32%, respectively. While, these paraments did not change significantly by the BL or Pyr treatments. Treatment with BL + Pyr and Pyr, rather than BL, improved the chlorophyll a and chlorophyll b contents by upregulating genes related to chlorophyll biosynthesis and downregulating genes related to chlorophyll degradation. Additionally, according to transcriptomic and metabolomic analysis, the BL+ Pyr treatment outperformed the individual BL or Pyr treatments in activating the transcription of genes involved in photosynthesis and increasing sugar accumulation. Our results first validated that the combined usage of BL and Pyr exerted striking synergistic effects on enhancing plant biomass and yield by increasing photosynthetic efficiency. These results might provide new understanding for the agricultural effects by the co-application of BL and Pyr, and it might stimulate the efforts to develop new environment-friendly replacement for Pyr to minimize the ecotoxicology of Pyr.

## 1 Introduction

Food security is a long-term and urgent issue (Godfray et al., 2010). Over the past decades, agricultural yields have risen, which is primarily due to the greater and more consistent crop production achieved by genetic engineering strategies (Bailey-Serres et al., 2019). However, the year-on-year increase in yields of major crops has plateaued in many parts of the world (Simkin et al., 2019). Additionally, the potential contribution of genetic modification engineering to yield increases were restricted, as the development of a new genetically engineered crop is a challenging, long-term, and expensive enterprise, and the application of genetically modified crops is severely limited in many countries (Prado et al., 2014). Currently, more than 800 million people are suffering from a food crisis globally (Wei et al., 2022). Moreover, the current crop yield increases are insufficient to feed nearly 10 billion people by 2050, which is even more concerning when the expected adverse effects of climate changes and the reduced availability of arable land are considered (Tilman et al., 2011; Araus et al., 2021).

Oxygenic photosynthesis initiates with light absorption, followed by excitation energy transfer to the reaction center, primary photochemistry, transport of electrons and protons, NADPH, and ATP synthesis, preceded by CO_2_ fixation through the Calvin–Benson cycle (Stirbet et al., 2020). Photosynthesis is regulated by various factors, such as the process of gas exchange, the content of photosynthetic pigments, the activity of photosynthesis-related enzymes, the transcription of photosynthesis-related genes, and the allocation of photosynthetic intermediates, *etc.* (Foyer and Noctor, 2000; Colombo et al., 2016; Heyneke and Fernie, 2018; Mu and Chen, 2021; Zahra et al., 2022). As photosynthetic products can either serve as carbon skeletons to assemble the whole plant or produce ATP through the re-oxidation by mitochondrial respiration to fuel metabolic or transport processes, optimization of photosynthesis has been demonstrated to be a practical approach for improving crop yield (Ruan, 2014; Nuccio et al., 2015). Numerous genes and enzymes involved in photosynthetic processes have been proven to be targeted for enhancing photosynthetic efficiency and thus yield (Long et al., 2015; Simkin et al., 2019; Araus et al., 2021).

Brassinosteroids (BRs), as the sixth class of plant hormones, regulate a broad spectrum of physiological functions in plants, such as cell division and elongation, xylem differentiation, photosynthesis, photomorphogenesis, senescence, and reproduction (Nolan et al., 2020). Furthermore, the physiological effects of BRs are more noticeable when the plant suffers from adverse environmental stress, leading BRs to protect against the pressures that plants may eventually experience (Sharma et al., 2018; Ahammed et al., 2020; Wang et al., 2020b). As reported, BRs and their analogs have been widely used as regulators of plant growth in agriculture to improve quality and yield (Vriet et al., 2012; Ali, 2017; Tong and Chu, 2018; Padhiary et al., 2020). However, the regulation of growth by endogenous BRs is temporally and spatially specific, and the effectiveness of exogenous BRs is susceptible to the time of application, treatment frequency, dose, and complex field conditions (Zu et al., 2019; Lin, 2020; Hwang et al., 2021). Therefore, the effects of exogenous BR application are not always as satisfactory as expected under field conditions due to the feedback inhibition or ineffectiveness of BR signaling, which has discouraged the usage of exogenous BRs in agriculture and horticulture (Khripach et al., 2000; Vriet et al., 2012).

Pyraclostrobin (Pyr), a strobilurin fungicide, is widely used in agriculture (Bartlett et al., 2002). Pyr not only shows fungicidal activity but also displays benefits for plant growth, including increasing net photosynthesis, improving the efficiency of water utilization, activating nitrate reductase, strengthening stress tolerance, and delaying senescence (Amaro et al., 2019). However, Pyr is more likely to improve plant biomass than grain yield due to the compensatory efforts of plants in adjusting yield components (Swoboda and Pedersen, 2009; Factor et al., 2010). Albeit with the beneficial regulatory functions, Pyr also exerts adverse effects on plants; for example, Pyr can partially inhibit electron transport in the cytochrome *bc1* complex of mitochondria, which leads to reduced ATP production in plants (Nason et al., 2007; Pedersen et al., 2017). Furthermore, Pyr also impaired the photosynthetic process due to the blockage of electron transport between photosystem II (PSII) and photosystem I (PSI) by binding to the Q_i_ site of the chloroplast cytochrome *bf* complex (Nason et al., 2007; Debona et al., 2016).

To alleviate the side effects of Pyr, Pyr in combination with BRs has gradually been used in the field. Interestingly, the combined use of BL and Pyr not only provided better protection in terms of reducing the phytotoxicity of Pyr but also produced unexpected beneficial improvements in plant growth in several field trials (Li et al., 2018; Li et al., 2020; Zhang, 2020; Li et al., 2021; Zhao et al., 2021). However, the abovementioned discovery of the benefits of BL and Pyr coapplication is still largely restricted to a few in-field applications. Since the threat of pests and pathogens is unavoidable in fields, it is quite difficult to distinguish the plant protective effect exerted by the intrinsic fungicidal activity of Pyr from the additional physiological benefits of the BR plus Pyr treatment on plants. Hence, the actual growth-promoting activity of BR plus Pyr coapplication through the preclusion of the fungicidal effect of Pyr, as well as the corresponding underlying mechanisms, remain to be experimentally verified in the laboratory.

For this purpose, the effects of different applications, including BL, Pyr, and BL+ Pyr, in *A. thaliana* were compared under pathogen-free environmental conditions. The results showed that, in addition to the benefits of disease control due to the fungicidal activity of Pyr, the coapplication of BL and Pyr exerted a synergistic effect on simultaneously enhancing the biomass and yield of *A. thaliana*. Based on the physiological, biochemical, transcriptomic and metabolomic analyses, we found that the synergistic enhancement of biomass and yield of BL+Pyr was related to the improved photosynthetic performance and the increased sugar accumulation. Our study indicated that applying a group of chemical compounds might be a simple but efficient way to promote plant productivity through the regulation of photosynthesis. Given that many reports indicated the adverse effects of Pyr on terrestrial and aquatic life forms (Liu et al., 2018; Zhang et al., 2020; Wang et al., 2020a), we hope that the identification of candidate genes and compounds based on transcriptome and metabolome analyses would be helpful to search and design new environmentally friendly yield-promoting agrochemicals.

## 2 Materials and methods

### 2.1 Plant material and experimental design

The surfaces of A. *thaliana* plant seeds (ecotype Col-0) were sterilized in ethanol-water (70:30, v/v) for 10 minutes, washed with distilled water and then plated on 0.5 × MS medium (Duchefa, Haarlem, the Netherlands) supplemented with 1% sucrose (Macklin, Shanghai) and 0.8% agar (Macklin, Shanghai). After being pretreated at 4°C for 2 days, the plates were placed horizontally in the artificial climate chamber under long-day conditions (22°C, 30% humidity, and approximately 120 photons μmol m^−2^ s^−1^ on a 16 h day/night cycle) until the seedlings were transplanted into the soil after 7 days.

The seedlings were grown for 14 days in soil, and then the seedlings with similar size were picked out for subsequent experiments. To determine the optimal concentration of BL+Pyr for promoting plant growth, seedlings were divided into 12 groups and each group included 10 biological replicates. Then the seedlings were sprayed with 1‰ DMSO (Sigma-Aldrich), 0.03, 0.3, 3 or 30 μM of pyraclostrobin (Pyr, Shanghai Lvze Bio-Tech Co. 99%), 0.1, 1 or 10 μM of brassinolide (BL, Sigma-Aldrich), 0.1, 1 or 10 μM of BL in combination with 0.03, 0.3, 3 or 30 μM of Pyr at each concentration, respectively. In the subsequent comprehensive study of the effects of BL (1 μM) and Pyr (3 μM) on plant growth, seedlings were divided into 4 groups and 60 biological replicates were used for each treatment. Then the seedings were separately foliar spray with 1‰ DMSO, BL (1 μM), Pyr (3 μM), or a mixture of BL (1 μM) and Pyr (3 μM). A total of 3 foliar sprays were applied to 20-day-old seedlings (vegetable growth stage), 35-day-old seedlings (floral development stage) and 55-day-old seedlings (silique development stage). The seedings treated with 1‰ DMSO was considered as untreated control group. The leaves of 31-day-old seedlings were harvested for further physiological, biochemical, transcriptomic and metabolomic analyses.

### 2.2 Phenotypic index measurement

To determine optimal concentration of BL+Pyr for promoting plant growth, the major axis and the fresh weight of the rosette leaves in each treatment were measured on 35-day-old seedlings with 10 biological replicates. 3 independent experiments were performed from October 2018 to December 2018.

In the comprehensive study of the effects of the coapplication of BL (1 μM) and Pyr (3 μM) on plant growth, the major axis and the fresh weight of the rosette leaves were measured on 27-day-old seedlings and 31-day-old seedlings with 20 biological replicates in each treatment. Similarly, 20 biological replicates in each treatment were measured for obtaining the fresh weights of rosette leaves and inflorescences of 39-day-old seedlings, 51-day-old seedlings, and 69-day-old seedlings or to count the inflorescence height, number of rosette branches, and number of branches with two or more seed-bearing siliques of 69-day-old seedlings. And the time of the first bud appearing and the first flower opening was recorded at 12-hour intervals with 60 biological replicates in each treatment. Also, 20 biological replicates in each treatment were used to collect the seeds every 5 days after the first silique shattered until senescence complete. Then the collected seeds were dried in an oven at 28°C for 48 hours before being weighed on a 1/10,000 scale to obtain the seed yield. Thousand kernel weight were weighed with 4 biological replicates in each treatment and calculated according to the following formula: Thousand kernel weight (mg) = weight (mg)/seed number × 1000. All the images were captured using a digital camera (Canon DS126201, Japan). Phenotypic and yield traits were assessed in the artificial climate chamber and 4 independent experiments were performed in Tianjin to obtain the results (March 2019 to July 2019, April 2019 to August 2019, June 2019 to October 2019, September 2019 to January 2020).

### 2.3 Measurement of rubisco activity

The *in-vitro* activity of ribulose-1,5-bisphosphate carboxylase/oxygenase (Rubisco; EC 4.1.1.39) was determined by monitoring NADH oxidation at 30°C at 340 nm, accompanied by the conversion of glycerol 3-phosphate to glycerol 3-phosphate upon the addition of an enzyme extract to the reaction mixture. Rubisco enzyme activity was measured by Rubisco Activity Assay Kit (Solarbio, China) according to the manufacturer’s instructions. Each independent experiment consisted of 3 biological replicates and 3 independent experiments were performed.

### 2.4 Measurement of chlorophyll content

Fresh leaf tissue (0.5 g) was homogenized on ice, extracted repeatedly with 80% acetone (v/v) until no visible pigment remained. The combined extracts were then centrifuged at 4500 g at 4 °C. The absorbance values of the solution at 646 and 663 nm were recorded to calculate the chlorophyll (Chl a and Chl b) contents (µg/g FW) according to a previous report (Lichtenthaler, 1987). Each independent experiment consisted of 3 biological replicates and 3 independent experiments were performed.


(1) $$C_{a}=\frac{12.21\times A_{663}-2.81\times A_{646}}{M_{plant}}$$

(2) $$C_{b}=\frac{20.13\times A_{646}-5.03\times A_{663}}{M_{plant}}$$

### 2.5 Measurement of photosynthesis-related indicators

Gas exchange parameters were measured on the 7^th^ leaf using an infrared gas analyzer (IRGA) portable photosynthesis system LI-6800 (Li-COR, USA) with a 2 cm² fluorescent leaf chamber. The gas exchange constants, including net photosynthetic rate (P_n_), transpiration rate (T_r_), stomatal conductance (G_s_), and intercellular CO_2_ concentrations (C_i_), were measured under the settings of 750 μmol s^−1^ flow rate, 600 μmol m^−2^ s^−1^ photosynthetic photon flux density (PPFD), and 400 ppm ambient CO_2_ (C_a_). During the measurement, leaf temperature and relative humidity were maintained at 22°C and at 55-65%, respectively. The stomatal restriction value (L_s_) and apparent mesophyll conductance (AMC) were calculated following the formulations L_s_=1-C_i_/C_a_ and AMC=P_n_/C_i_. Each independent experiment consisted of 10 biological replicates and 3 independent experiments were performed.

The light response curves and the CO_2_ response curves were also measured on the 7^th^ leaf according to the standard settings of the Li-COR 6800’s automatic procedure. Saturated light intensity (PARsat) were calculated with a modified model of the light reaction curve for plant photosynthesis (Ye and Kang, 2012). The dark respiration rate (Rd) and the apparent quantum efficiency (AYE) were calculated according to the previous reports (Sekhar et al., 2014; Sekhar et al., 2015). The V_cmax_, V_TPU_ and J_max_ were calculated according to Farquhar, von Caemmerer and Berry model using PCE Calculator(version 2.0) (Long and Bernacchi, 2003). Each independent experiment consisted of 2 biological replicates and 3 independent experiments were performed.

Chlorophyll fluorescence parameters were measured on the 7^th^ leaf. Plants were dark treated for 1 hour prior to the measurement of the minimal fluorescence (F_o_), after which the maximum fluorescence (F_m_) was measured by irradiating a polyphasic saturating flash (4000 μmol m^-2^ s^-1^). Subsequently, the steady-state fluorescence under light (F_s_), the maximum fluorescence under light (F_m_’) and the minimum fluorescence under light (F_o_’) were recorded on the leaves being adequately light-adapted for 1 hour. The quantum yield of PSII (ΦPSII), maximum quantum yield of PSII (F_v_/F_m_), efficiency of energy capture by open PSII (F_v_’/F_m_’), electron transfer rates (ETR) were calculated as previously described (Maxwell and Johnson, 2000; Sreeharsha et al., 2015). Each independent experiment consisted of 10 biological replicates and 3 independent experiments were performed.

### 2.6 Extraction of mRNA and real-time fluorescence PCR (qRT-PCR)

Leaf samples (0.1 g) were ground in liquid nitrogen, and total RNA was extracted using an RNA extraction kit (TransGen, China) according to the instructions. The extracted RNAs were then converted to cDNA using a PrimScript RT kit (Takara, Kyoto, Japan). 10 genes (*PSAB*, *PSAA*, *PSAF*, *PSBA*, *ATPD*, *CPN60A1*, *RBCL*, *RCA*, *SBPASE*, *CFBP*) were selected for the validation of Seq-analysis. The expression levels of the target genes were analyzed by qRT-PCR using *Actin 2* as the internal reference gene and using SYBR Premix Ex TaqTM (Takara, Kyoto, Japan) as the fluorescent dye. The primers for the target genes were listed in **Table S1**. The relative expression of the target genes was calculated according to the 2^-ΔΔCT^ formula. Each independent experiment consisted of 3 biological replicates and 3 independent experiments were performed to obtain the results.

### 2.7 RNA-seq analysis

RNA-Seq analysis was conducted using the UMI-mRNA sequencing method by Novogene Bioinformatics Technology Co., Ltd. (Tianjin, China). The mRNA libraries were prepared and sequenced on an Illumina Nova Seq 6000 platform with 3 biological replicates. After filtering the raw data, clean data with high quality were subjected to subsequent analysis. Then, FPKM (expected number of fragments per kilobase of transcript sequence per million base pairs sequenced) values were used to estimate gene expression levels. Differential expression analysis was performed using the DESeq R package (1.10.1), and hypothesis test probability (p-value) was corrected using the Benjamin−Hochberg method. Genes with an FDR (false discovery rate)<0.05 were considered differentially expressed. GO and KEGG enrichment analyses of differentially expressed genes were conducted using the Gene Ontology (http://geneontology.org/) and KEGG PATHWAY (https://www.kegg.jp/kegg/pathway.html) databases, respectively. All raw sequence data from this study have been deposited into the NCBI’s SRA database with the link of https://www.ncbi.nlm.nih.gov/sra/PRJNA930055, under the accession number SAMN32982539 to SAMN32982550, the temporary Submission ID SUB12691837 and the BioProject ID PRJNA930055.

### 2.8 Metabolite profiling

Metabolite profiling was carried out using the Quasi-Targeted Metabolomics method by Novogene Bioinformatics Technology Co., Ltd. (Tianjin, China) with 3 biological replicates. Metabolites were extracted according to an available protocol(Want et al., 2013), and untargeted metabolites were screened by LC-MS/MS analyses were performed using an ExionLC™ AD system (SCIEX) coupled with a TRAP^®^ 6500^+^ mass spectrometer (SCIEX). After metabolites were detected using MRM (multiple reaction monitoring) based on the Novogene in-house database, metabolite quantification and identification were performed using Q3 and Q1, Q3, RT (retention time), DP (depolymerization potential) and CE (collision energy). The integration and correction of peaks in data files generated by HPLC−MS/MS were performed using SCIEX OS version 1.4. The statistical significance (P-value) was calculated based on univariate analysis (T-test), and the metabolites with variable importance in projection (VIP) ≥ 1, fold change (FC) ≤ 0.8 or ≥1.2, and P-value< 0.05 were identified as DAMs (differential accumulation metabolites). These metabolites were annotated using the online KEGG (http://www.kegg.jp/), HMDB (http://www.hmdb.ca/) and Lipidmaps (http://www.lipidmaps.org/) databases.

### 2.9 Statistical analysis

To compare the differences among different treatment groups, statistical analysis was performed with one-way ANOVA followed by Tukey’s HSD test (*P*< 0.05) using SPSS software (Ver 26.0, Chicago, IL, USA). The data are presented as the means ± standard deviations (SD).

## 3 Results

### 3.1 The increase in plant biomass and seed yield of *A. thaliana* seedlings by the coapplication of brassinolide and pyraclostrobin

To explore the optimal concentration of BL + Pyr for promoting plant growth, the main axis and fresh weight of rosette leaves of *A. thaliana* treated with different concentrations of BL, Pyr and BL + Pyr were measured separately. As shown in **Figure S1**, no significant changes in leaf growth were observed at BL concentrations of 0.1 or 1 μM, while abnormal elongation of the rosette with an increased major axis but reduced fresh weight was observed for plants treated with 10 μM BL (**Figure S1A, B**). Similarly, Pyr promoted leaf growth at low concentrations (0.3 μM) and inhibited leaf growth at high concentrations (30 μM) (**Figures S1C, D**). There was no significant effect when the applied concentration of Pyr was 0.03 or 3 μM (**Figures S1C, D**). When different concentrations of BL (0.1 μM, 1 μM, 10 μM) or Pyr (0.03 μM, 0.3 μM, 3 μM, 30 μM) were coapplied, the coapplication of BL (1 μM) plus Pyr (3 μM) outperformed all other treatments in terms of simultaneously increasing the major axis and fresh weight of the rosette (**Table S2, S3**). In contrast, 1 μM BL or 3 μM Pyr induced no significant effect on the major axis and fresh weight of the rosette (**Table S2, S3**).

**Figure 1:**
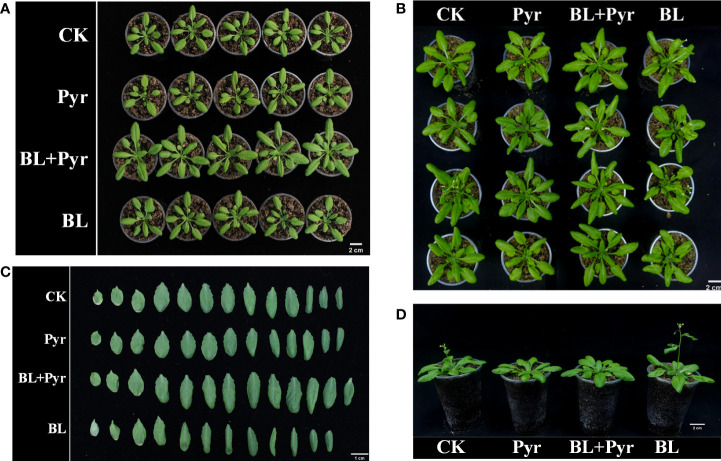
BL+Pyr increased the major axis and the fresh weight of rosette leaves during the vegetative growth period. Photos were taken on the 7^th^ day after the first-round application (**A**, 27-day-old seedlings) and 11^th^ day after the first-round application (**B–D**, 31-day-old seedlings). CK, untreated seedlings; Pyr, seedlings treated with 3 μM pyraclostrobin; BL+Pyr, seedlings treated with 1 μM BL and 3 μM pyraclostrobin; BL, seedlings treated with 1 μM BL.

To comprehensively study the synergistic effect of BL and Pyr on plant growth, BL (1 μM), Pyr (3 μM) and BL (1 μM) + Pyr (3 μM) were chosen, and the phenotypic traits were assessed throughout the vegetative stage and reproductive stage. In the vegetative stage, a significant effect on leaf growth of 27-day-old seedlings was observed only in the BL + Pyr group rather than the BL or Pyr group, with the major axis and the fresh weight of rosette leaves increasing by 21% and 41.5% compared with the untreated group, respectively (**Table 1**, **Figure 1A**). Similarly, only the BL + Pyr treatment increased the major axis and the fresh weight of rosette leaves of 31-day-old seedlings by 20.3% and 30.3% compared with the untreated group, respectively. In contrast, treatment with BL alone decreased the fresh weight of leaves by 17.8% compared to the untreated group (**Table 1**, **Figures 1B-D**).

**Table 1:** The major axis (cm) and fresh weight (mg) of rosette leaves during vegetative development stage.

Paraments	Age (days)	Pesticide Applied strategy			
		CK	Pyr	BL+Pyr	BL
Major axis(cm)	27	8.21 ± 0.55b	8.28 ± 0.63b	9.96 ± 0.53a	8.59 ± 0.67b
	31	9.5 ± 0.68b	9.74 ± 0.64b	11.49 ± 0.93a	10.02 ± 0.58b
Fresh weight(mg)	27	373 **±** 26b	396 **±** 22b	529 ± 42a	382 **±** 29b
	31	800 **±** 75b	828 **±** 65b	1048 ± 95a	668 **±** 51c

Data was measured on the 7^th^ day after the first-round application (27-day-old seedlings) and 11^th^ day after the first-round application (31-day-old seedlings). Data were presented as the mean ± SD of four independent replicate experiments. In a line, different letters indicate significant differences (p< 0.05) according to ANOVA followed by Tukey’s test. CK, untreated seedlings; Pyr, Treated with 3 μM pyraclostrobin; BL+Pyr, Treated with 1 μM BL in combination with 3 μM pyraclostrobin; BL, Treated with 1 μM BL.

**Figure 2:**
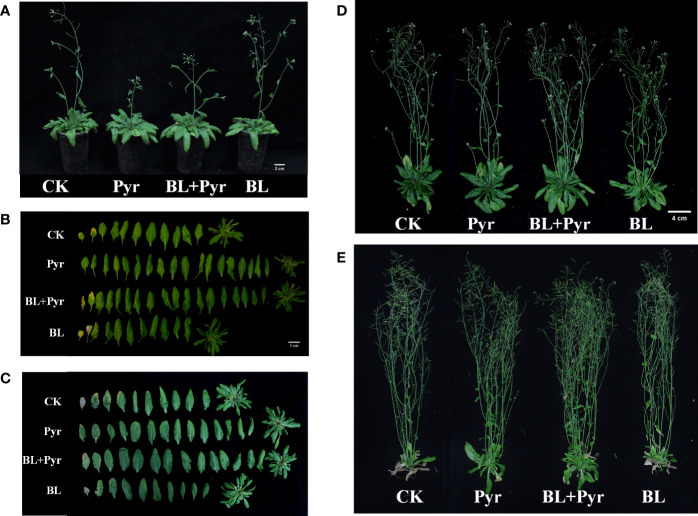
BL+Pyr increased the major axis and the fresh weight of rosette leaves and inflorescence during the reproductive growth period. Photos were taken on the 4^th^ day after the second-round application (**A, B**, 39-day-old seedlings), 16^th^ day after the second-round application (**C**, **D**, 51-day-old seedlings), and 14^th^ day after the third-round application (**E**, 69-day-old seedlings). CK, untreated seedlings; Pyr, seedlings treated with 3 μM pyraclostrobin; BL+Pyr, seedlings treated with 1 μM BL and 3 μM pyraclostrobin; BL, seedlings treated with 1 μM BL.

**Figure 3:**
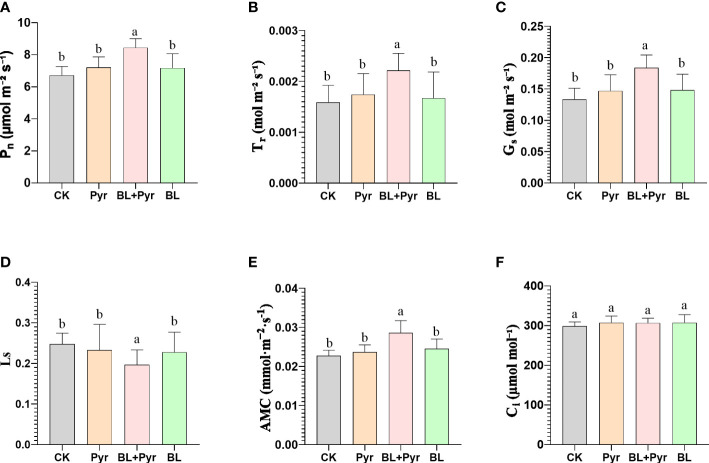
BL+Pyr showed a synergistic effect on increasing the photosynthetic efficiency by enhancing the gas exchange process. **(A)** Net photosynthetic rate (P_n_); **(B)** Transpiration rate (T_r_); **(C)** Stomatal conductance (G_s_); **(D)** Stomatal restriction value (L_s_); **(E)** Apparent mesophyll conductance (AMC); **(F)** Intercellular CO_2_ concentrations (C_i_). Data are presented as the mean ± SD of three independent replicate experiments. Different letters indicate significant differences (p< 0.05) according to ANOVA followed by Tukey’s test. 31-day-old seedlings (the 11^th^ day after the first-round application) were used. CK, untreated seedlings; Pyr, seedlings treated with 3 μM pyraclostrobin; BL+Pyr, seedlings treated with 1 μM BL and 3 μM pyraclostrobin; BL, seedlings treated with 1 μM BL.

In addition to comparing the traits of leaf growth in the vegetative stage, the phenotypic traits of four groups (untreated, BL+Pyr-treated, BL-treated or Pyr-treated) were further compared during the reproductive development stage. In the early stage of reproductive development (39-day-old seedlings), the fresh weight of rosette leaves increased by 51% and 42% when treated with BL+Pyr and Pyr, respectively, and decreased by 11% when treated with BL compared to the untreated group. Conversely, the fresh weight of inflorescences decreased by 43% and 74% when treated with BL+Pyr and Pyr, respectively, and increased by 18% when treated with BL compared to the untreated group (**Table 2**, **Figures 2A**, **2B**). Furthermore, the budding time and the corresponding flowering time were accelerated by the BL treatment and delayed by both the BL+Pyr and Pyr treatments compared with the untreated group (**Figures 2A**, **3A-B**).

**Table 2:** The fresh weight (mg) of rosette leaves and inflorescence during the reproductive development stage.

Paraments	Age (days)	Pesticide Applied strategy			
		CK	Pyr	BL+Pyr	BL
Fresh weight of leaves	39	1513 **±** 108b	2147 **±** 187a	2286 **±** 175a	1336 **±** 144c
	51	2776 ± 432b	3377 ± 579a	3490 ± 636a	2179 ± 264c
	69	285 ± 29c	1122 ± 118a	803 ± 114b	231 ± 23d
Fresh weight of inflorescence	39	1121 **±** 109b	291 **±** 138d	633 **±** 34c	1326 **±** 187a
	51	2941 ± 323b	2107 ± 507c	4123 ± 579a	3685 ± 495a
	69	3988 **±** 833c	4147 **±** 609c	5828 **±** 1026a	4946 **±** 724b

Data was measured on the 4^th^ day after the second-round application (39-day-old seedlings), the 16^th^ day after the second-round application (51-day-old seedlings), and the 14^th^ day of the third-round application (69-day-old seedlings). Data were presented as the mean ± SD of four independent replicate experiments. In a line, different letters indicate significant differences (p< 0.05) according to ANOVA followed by Tukey’s test. CK, untreated; Pyr, Treated with 3 μM pyraclostrobin; BL+Pyr, Treated with 1 μM BL in combination with 3 μM pyraclostrobin; BL, Treated with 1 μM BL.

In the mid-stage of reproductive development (51-day-old seedlings), the fresh weight of rosette leaves and inflorescence in the group treated with BL+Pyr were increased by 25% and 40%, respectively, compared to the untreated group (**Table 2** and **Figures 2C, D**). The fresh weight of rosette leaves was increased by 21% in the group treated with Pyr and decreased by 22% in the group treated with BL. Conversely, the fresh weight of inflorescence was decreased by 28% in the group treated with Pyr and increased by 25% in the group treated with BL compared to the untreated group (**Table 2**, **Figures 2C, D**). This change was also observed in the late stage of reproductive development (69-day-old seedlings). Compared to the untreated group, the fresh weight of rosette leaves and inflorescence was significantly increased by 181% and 46% in the BL+Pyr group, while Pyr treatment increased the fresh weight of rosette leaves by 293% without any obvious influence on the fresh weight of the inflorescence. In contrast, BL treatment increased the fresh weight of inflorescence by 24% and showed no detectable effect on the fresh weight of rosette leaves (**Table 2**, **Figure 2E**).

To explore the reasons for the variation in the fresh weight of inflorescences among the four groups, measurements were subsequently performed on the height of inflorescence, the number of rosette branches and effective branches in the late stage of reproductive development. No significant difference in inflorescence height was observed among the four groups (**Table 3**). Consistent with the fresh weight of inflorescence, the seedlings treated with BL+Pyr generated the most rosette branches and effective branches (branches with two or more seed-bearing siliques), followed by the seedlings treated with BL, then the untreated seedlings, and finally, the seedlings treated with Pyr (**Figures 2D-E**, **Table 3**).

**Table 3:** The floral development, inflorescence architecture, and seed yield parameters.

	Pesticide Applied strategy			
Parament	CK	Pyr	BL+Pyr	BL
The first bud appearing time (day)	30.37 ± 1.30c	32.60 ± 1.34a	31.43 ± 1.13b	29.23 ± 1.25d
The first flower opening time (day)	33.16 ± 1.56c	34.83 ± 1.64a	33.94 ± 1.29b	31.8 ± 1.36d
Number of Rosette Branches	5.03 ± 0.91b	4.55 ± 0.82c	6.12 ± 0.92a	5.42 ± 1.05b
Number of Effective Branches	23.5 ± 3.5c	22.3 ± 3.9c	30.8 ± 4.5a	26.2 ± 3.2b
Length of branch (cm)	32.21 ± 2.48	31.79 ± 2.50	31.02 ± 2.68	32.66 ± 3.06
Seeds weight per plant (mg)	239.6 ± 32.5c	252.6 ± 30.2bc	301.2 ± 51.0a	263.1 ± 31.1b
Thousand kernel weight (mg)	19.03 ± 0.62	19.28 ± 1.40	19.12 ± 1.08	19.29 ± 0.74

The first bud appearing time (n=60)and the first flower opening time (n=60) was counted at 12 hourly intervals during the floral transition and opening stage; The height of inflorescence(cm, n=60), number of rosette branches (n=60) and number of branches with two or more seeds-bearing siliques (n=60) were measured on 14^th^ day after the third-round application (69-day-old seedings); Seeds weight per plant (n=60, mg) and thousand kernel weight (n=12, mg) were measured after dried in an oven at 28°C for 48 hours. Data are presented as the mean ± SD of four independent replicate experiments. Different letters indicate significant differences (p< 0.05) according to ANOVA followed by Tukey’s test. CK, untreated; Pyr, Treated with 3 μM pyraclostrobin; BL+Pyr, Treated with 1 μM BL in combination with 3 μM pyraclostrobin; BL, Treated with 1 μM BL.

Finally, the seed yield per plant and thousand-kernel weight of the 4 groups (untreated, BL+Pyr, BL and Pyr) were further compared. Compared to the untreated group, the seed yield was increased by 25.6% in the BL+Pyr group, which was much higher than that (9.7%) in the BL group. In contrast, Pyr did not show a significant increase in seed yield (**Table 3**). Additionally, there was no significant difference in thousand-kernel weight among the four groups (**Table 3**).

### 3.2 Synergistic enhancement of photosynthetic efficiency by the coapplication of brassinolide and pyraclostrobin

To investigate whether the mechanism by which the BL + Pyr treatment synergistically enhanced the biomass and yield was related to photosynthesis, the influence of the four treatments (untreated, BL+Pyr, BL and Pyr) on photosynthetic traits was analyzed. The results of the gas exchange constant measurements indicated that BL+Pyr treatment significantly increased the net photosynthetic rate (P_n_), the stomatal conductance (G_s_), the transpiration rate (T_r_), and the apparent mesophyll conductance (AMC) by 26%, 38%, 40% and 25%, respectively, while decreased the stomatal restriction value (L_s_) by 20%, compared to the untreated group (**Figures 3A-E**). While, no statistically significant differences were observed between the untreated group and individual BL or Pyr treatments regarding these above gas exchange parameters (**Figure 3**). However, the enhanced gas exchange process in the BL + Pyr treatment did not result in a corresponding increase in intercellular CO_2_ concentration (C_i_), as C_i_ showed almost no change between the four treatments (untreated, BL + Pyr, BL and Pyr) (**Figure 3F**).

**Figure 4:**
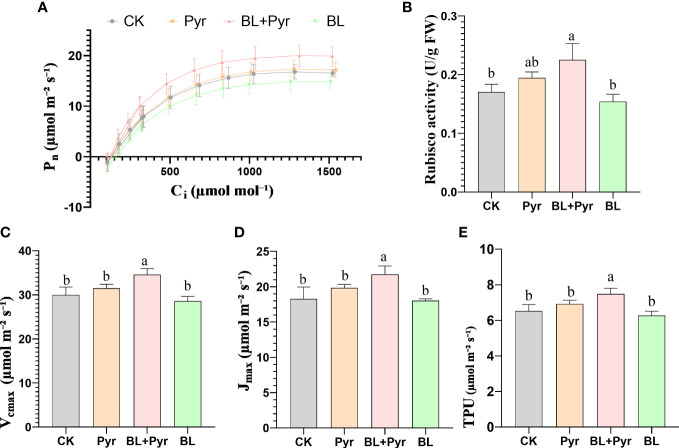
BL+Pyr showed a synergistic effect on increasing CO_2_ assimilation efficiency. **(A)** Net photosynthetic rate (P_n_) at different CO_2_ concentration (400, 300, 200, 100, 400, 400, 600, 800, 1000, 1200, 1500, 1800 µmol mol⁻¹) under saturated light intensity (600 µmol m⁻² s⁻¹); **(B)** Rubisco enzyme activity (U/g FW); **(C)** Maximum *in-vivo* Rubisco carboxylation rates (V_c,max_); **(D)** the maximum rate of electron transport driving regeneration of RuBP (J_max_); **(E)** Maximum rate of photosynthetic product triose-phosphate utilization (V_TPU_. Data are presented as the mean ± SD (n=3, measured at random in 3 separate replicate experiments). Data are presented as the mean ± SD of three independent replicate experiments. Different letters indicate significant differences (p< 0.05) according to ANOVA followed by Tukey’s test. 31-day-old seedlings (the 11^th^ day after the first-round application) were used. CK, untreated seedlings; Pyr, seedlings treated with 3 μM pyraclostrobin; BL+Pyr, seedlings treated with 1 μM BL and 3 μM pyraclostrobin; BL, seedlings treated with 1 μM BL.

To compare CO_2_ assimilation efficiency among the four groups, CO_2_ response curves were measured. As shown in **Figure 4**, the ribulose-1,5-bisphosphate (RuBP) carboxylase/oxygenase (Rubisco) carboxylation (V_cmax_), the maximum rate of the electron transport driving regeneration of RuBP (J_max_) and the triose-phosphate utilization (V_TPU_) were improved by 16%, 19% and 15%, respectively, in the group treated with BL + Pyr, while they were not significantly changed in the groups treated with BL or Pyr alone compared to the untreated group (**Figure 4**).

As the increase in CO_2_ assimilation efficiency was largely correlated with Rubisco enzyme activity, Rubisco enzyme activity was then compared. Consistent with the increase in CO_2_ assimilation efficiency, ribulose-1,5-bisphosphate carboxylase/oxygenase (Rubisco) enzyme activity was also increased by 32% in the BL+Pyr group, while no significant increase was identified in the BL or Pyr-treated groups in comparison with the untreated group (**Figure 4B**).

To verify that the differences in photosynthetic characteristics were not associated with the changes in individual saturation light intensity between the four groups, light response curves were generated. The results verified that saturated light intensity (L_sat_) did not greatly vary among the four groups (**Figure S2**). Furthermore, the BL+Pyr treatment improved the dark respiration rate (Rd) and the apparent quantum efficiency (AYE) by 179% and 18%, respectively, when compared to the untreated group (**Figure S3**). While, no significant increase of Rd and AYE was identified in the BL or Pyr-treated groups in comparison with the untreated group (**Figure S3**).

To better understand the light absorption, electron transfer, and energy partitioning in photosynthetic apparatus in the four groups, chlorophyll fluorescence parameters were further measured. As shown in **Figure S4**, the efficiency of excitation energy capture by open PSII (F_v_’/F_m_’), the actual quantum yield of PSII (ΦPSII) and the electron transfer rates (ETR) increased by 10%, 9% and 17% in the group treated with BL+Pyr, while did not significantly varied in the BL or Pyr-treated groups, when compared to the untreated group (**Figure S4**). Meanwhile, there was no significant difference in the maximum quantum yield of PSII (F_v_/F_m_) among the four groups (**Figure S4**).

### 3.3 The positive regulation of chlorophyll synthesis by the coapplication of brassinolide and pyraclostrobin

**Figure 5:**
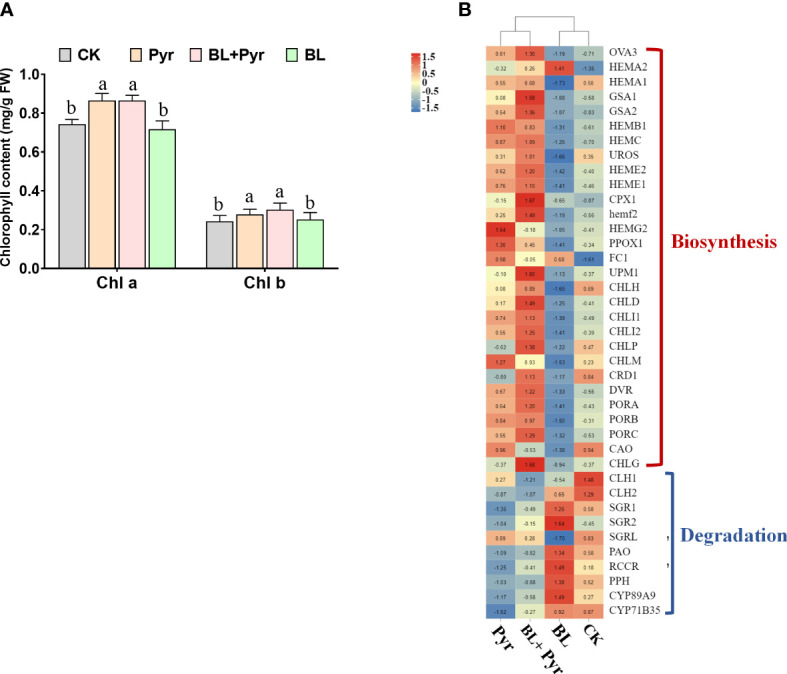
BL+Pyr and Pyr increased chlorophyll contents by regulating the transcript levels of genes in the chlorophyll metabolism pathway. **(A)** The chlorophyll a and chlorophyll b contents (mg/g FW); **(B)** Hierarchical cluster analysis of the differentially expressed genes (DEGs) associated with chlorophyll biosynthesis and degradation, and FPKM was Z-scole normalised prior to analysis. Data are presented as the mean ± SD of three independent biological replicates. Different letters indicated significant differences (p< 0.05) according to ANOVA followed by Tukey’s test. The red line represents positive correlation and the blue line represents negative correlation. 31-day-old seedlings (the 11^th^ day after the first-round application) were used. CK, untreated seedlings; Pyr, seedlings treated with 3 μM pyraclostrobin; BL+Pyr, seedlings treated with 1 μM BL and 3 μM pyraclostrobin; BL, seedlings treated with 1 μM BL.

To determine the unique role of chlorophyll content in the improvement of photosynthetic performance by the BL + Pyr treatment, the contents of chlorophyll a and chlorophyll b in the 4 groups were further compared. Compared with the untreated group, the contents of chlorophyll a and chlorophyll b were substantially increased by 16% and 26% in the BL+Pyr group, respectively, which was similar to the percentage of increase in the Pyr group (**Figure 5A**). However, there was no obvious change between the BL treatment and untreated groups (**Figure 5A**).

To explore the mechanisms underlying the variation in chlorophyll content among the four groups, the differentially expressed genes (DEGs) involved in the chlorophyll metabolic pathway were further analyzed according to the transcriptome analysis. As shown in **Figure 5B**, the majority of genes associated with chlorophyll biosynthesis, such as the genes encoding protoporphyrinogen oxidase (*PPO*), tetrapyrrole (corrin/porphyrin) methylases (*UMP1*), Mg chelatase (*CHLD, CHLI1, CHLI2*), magnesium-protoporphyrin IX methyltransferase (*CHLM*), 3,8-divinyl protochlorophyllide a 8-vinyl reductase (*DVR*), protochlorophyllide oxidoreductase (*PORA, PORB, PORC*), and Chl synthase (*CHLG*), were upregulated in both the BL+Pyr and Pyr groups. Conversely, the majority of genes associated with chlorophyll degradation, such as the genes encoding chlorophyllase (*CLH1* and *CLH2*), pheophorbide a oxygenase (*PAO*), chlorophyll b reductase (*NYC1*), RCC reductase (*RCCR*), pheophytinase (*PPH*), and Mendel’s Stay-green gene (*SGR1*), which encodes Mg-dechelatase, were downregulated in both the BL+Pyr and Pyr groups. In contrast to these two treatments, the transcript levels of several chlorophyll biosynthesis-related genes were downregulated and several chlorophyll degradation-related genes were upregulated by BL treatment.

### 3.4 RNA-Seq reveals the improved transcription levels of photosynthesis-related genes by the coapplication of brassinolide and pyraclostrobin

**Figure 6:**
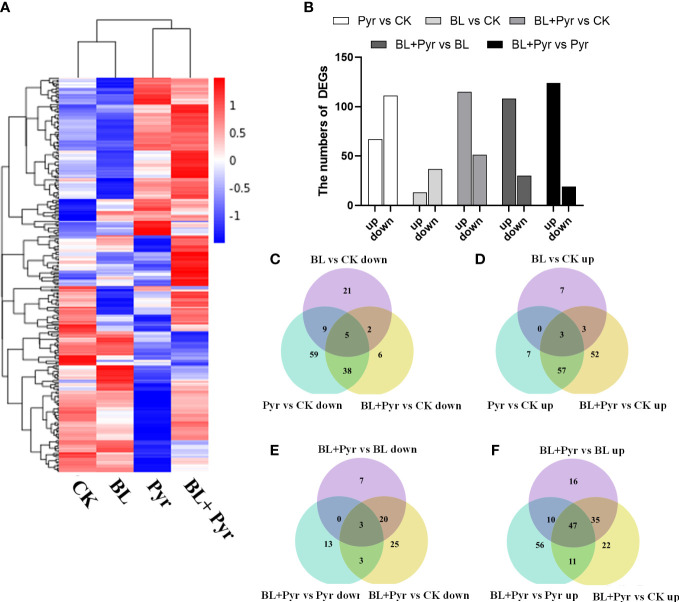
BL+Pyr activated the transcription of genes related to photosynthesis. **(A)** Hierarchical cluster analysis of the differentially expressed genes (DEGs) involved in photosynthesis according to the average FPKM (expected number of Fragments Per Kilobase of transcript sequence per Millions base pairs sequenced) of 3 biological repeats; **(B)** The number of photosynthesis-related DEGs among the comparisons. ‘up’ represented up-regulated and ‘down’ represented down-regulated. **(C-F)** Venn diagrams showing the overlapping and non-overlapping (DEGs) related to photosynthesis among the comparisons. The genes with an FDR (the false discovery rate)<0.05 were assigned as DEG. CK, untreated; Pyr, Treated with 3 μM pyraclostrobin; BL+Pyr, Treated with 1 μM BL in combinations with 3 μM pyraclostrobin; BL, Treated with 1 μM BL.

To gain a deeper understanding of the transcriptional mechanisms by which the BL+Pyr treatment specifically enhances photosynthetic performance, the photosynthesis-related DEGs were analyzed based on the transcriptome analysis of the four treatment groups (untreated, BL+Pyr, BL and Pyr). The results showed that BL+Pyr treatment (51 downregulated and 115 upregulated) and Pyr treatment (111 downregulated and 67 upregulated) both greatly altered the expression levels of genes involved in photosynthesis, followed by BL treatment (37 downregulated and 13 upregulated), which had a relatively weak effect on those genes, compared with the untreated group (**Figures 6A, B**). In addition, the BL+Pyr treatment also resulted in remarkable differences in the expression levels of photosynthesis-related genes relative to the BL or Pyr treatment, with 124 upregulated and 19 downregulated DEGs relative to the Pyr treatment and 108 upregulated and 30 downregulated DEGs relative to the BL treatment (**Figures 6A, B**). To validate the RNA-seq data, 10 photosynthesis-related DEGs were selected for qRT−PCR validation. The differential expression of these genes according to qRT−PCR was highly correlated with the RNA-seq data, confirming the transcriptome data (**Figure S5**).

**Figure 7:**
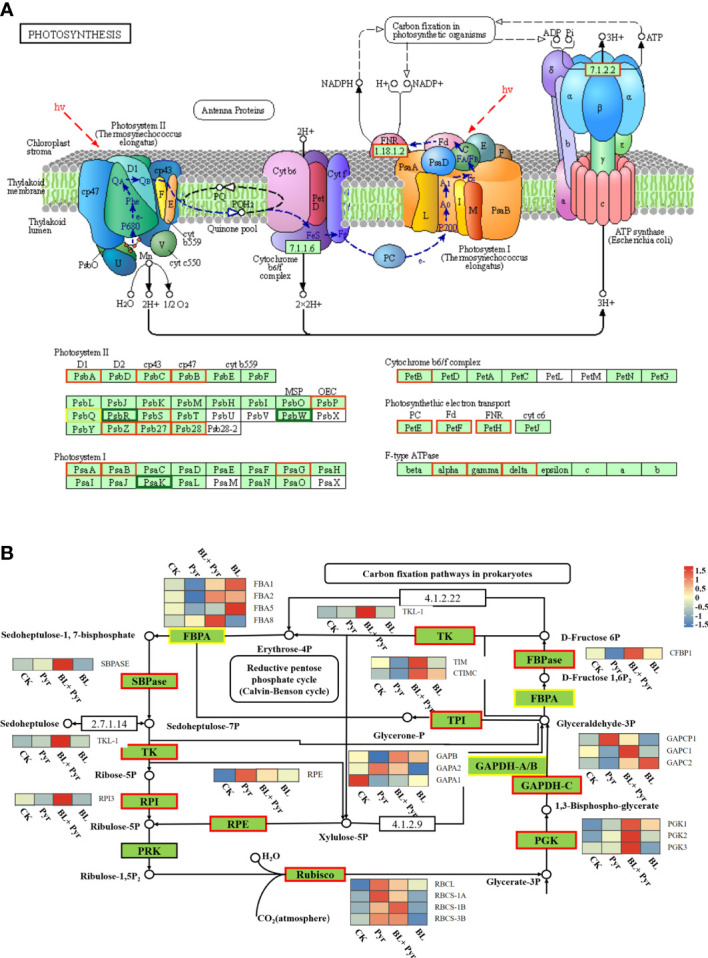
BL+Pyr treatment activated transcription of genes in photosynthesis and carbon fixation pathway. **(A)** Photosynthesis pathway tagged with DEGs of BL+Pyr-treated group versus untreated group; **(B)** Carbon fixation pathway tagged with DEGs of BL+Pyr-treated group versus untreated group. The heat map close to the enzyme showed the expression level of the gene encoding the corresponding enzyme. The different colored boxes on the protein names indicate that the gene encoding the protein is either up- or down-regulated by the BL+Pyr-treated group versus the untreated group. CK, untreated; Pyr, Treated with 3 μM pyraclostrobin; BL+Pyr, Treated with 1 μM BL in combinations with 3 μM pyraclostrobin; BL, Treated with 1 μM BL. SBPase, Sedoheptulose-1,7-bisphosphatase; FBPA, Fructose-1,6-bisphosphate aldolase; FBPase, Fructose-1,6-bisphosphatases; TK, Transketolase; RPE, Ribulose-phosphate 3-epimerase; TPI, Triosephosphate isomerase; GAPDH, Glyceraldehyde 3-phosphate dehydrogenase; PGK, Phosphoglycerate kinase; RPI, Ribose 5-phosphate isomerase A; PRK, Phosphoribulokinase.

Further analysis of the expression profiles differentially expressed gene in the photosynthetic and carbon fixation pathways was performed. The results showed that the transcription levels of the key proteins involved in the photosynthesis pathway, such as PSII (*PSBA*, *PSBB*, *PSBC*, *etc.*), PSI (*PSAA* and *PSAB, etc.*), ferredoxin (*FD2* and *FdC1*), plastocyanin (*DRT112*), Cyt *b_6_f* (*PETB*), and ATP synthase (*ATPA*, *ATPC* and *ATPD*), were increased by the BL+Pyr treatment (**Figures 7A**, **Table S4**). Additionally, the transcription levels of the majority of enzymes in the Calvin cycle, including Rubisco, sedoheptulose-1,7-bisphosphatase (SBPase), fructose-1,6-bisphosphate aldolase (FBPA), fructose-1,6-bisphosphatases (FBPase), transketolase (TK), ribulose-phosphate 3-epimerase (RPE), triosephosphate isomerase (TPI), glyceraldehyde 3-phosphate dehydrogenase (GAPDH), phosphoglycerate kinase (PGK), and ribose 5-phosphate isomerase A (RPI), were also increased by the BL+Pyr treatment (**Figure 7B**, **Table S4**). In contrast, Pyr treatment and BL treatment showed opposite effects in regulating the transcript levels of genes in the photosynthetic light reaction pathway and carbon fixation pathway. Pyr treatment upregulated the transcript levels of the reaction center proteins PSII and PSI and downregulated the transcript levels of the majority of enzymes in the Calvin cycle (**Figures S6A-B**). Conversely, BL treatment downregulated the transcript levels of several PSII and PSI subunit proteins and upregulated the transcript levels of major enzymes in the Calvin cycle (**Figures S6C-D**).

To discriminate hub genes related to photosynthesis whose expression was induced by the BL+Pyr treatment, the photosynthesis-related DEGs uniquely regulated by the BL+Pyr treatment were further analyzed, including the nonoverlapping photosynthesis-related DEGs between the BL+Pyr treatment and BL or Pyr treatments versus the untreated group and the overlapping photosynthesis-related DEGs between the BL+Pyr group and the other 3 groups (untreated, BL and Pyr groups) (**Figures 6C-F**). A total of 79 DEGs fit the above criteria, including 10 downregulated and 69 upregulated DEGs (**Figure S7** and **Table S5**). To investigate the specific functions and pathways of photosynthesis that were uniquely transcriptionally regulated by BL+Pyr treatment, GO and KEGG enrichment analyses were performed on those 79 DEGs. The significantly enriched GO terms were photosynthesis, generation of precursor metabolites and energy, photosynthesis-light reaction, carbon fixation, hexose biosynthetic process, and photosynthetic electron transport chain, and more related DEGs were upregulated than downregulated (**Figure S8**). Similarly, the significantly enriched KEGG pathways were photosynthesis and carbon fixation in photosynthetic organisms, and all of the related DEGs were upregulated (**Figure S8B**). In addition, the in-depth analysis of these 79 genes showed that the majority of the genes that were specifically upregulated by BL+Pyr were associated with the assembly factors of PSII and PSI, photosynthetic electron transport and the key enzyme of the Calvin cycle (**Table S5**).

### 3.5 The metabolome analysis validates the increased accumulation of photosynthates by the coapplication of brassinolide and pyraclostrobin

**Figure 8:**
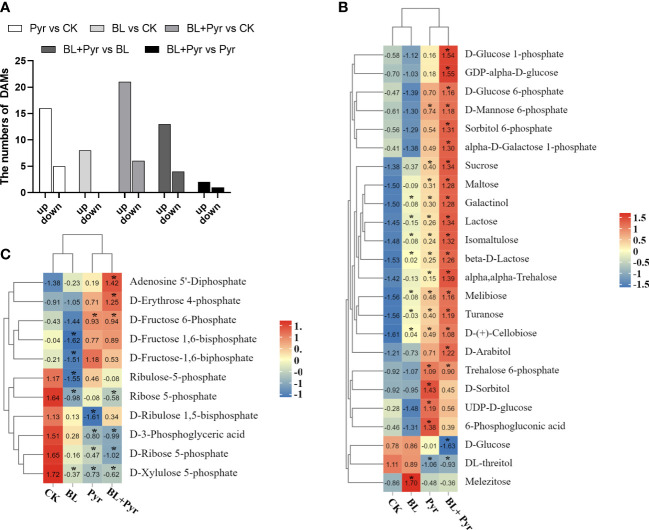
BL+Pyr showed a synergistic effect on increasing the accumulation of photosynthates. **(A)** Number of DAMs in the CO_2_ fixation pathway and belonging to sugars of the three treatments (BL + Pyr, BL and Pyr) versus the untreated group; **(B)** Hierarchical cluster analysis of the sugars content of the three treatments (BL + Pyr, BL and Pyr) versus the untreated group; **(C)** Hierarchical cluster analysis of the content of DAMs in the CO_2_ fixation pathway of the three treatments (BL + Pyr, BL and Pyr) versus the untreated group. The Metabolites with variable importance in projection (VIP) ≥ 1, fold change (FC) ≤ 0.8 or ≥1.2, and P-value< 0.05 were classified as DAMs (differential accumulation metabolites). Values shown in **(B, C)** are Z-scores normalized to concentrations, and ‘*’ represent significant differences versus the untreated group.

Since the co-application of BL and Pyr promoted photosynthesis efficiency by regulating multiple genes, we then used metabolome analysis to identify the variations in the accumulation of photosynthates in the four groups. As shown in **Figure 8A**, a total of 35 DAMs in the CO_2_ fixation pathway and belonging to sugars were identified in the three groups (BL + Pyr, BL and Pyr) versus the untreated group, including 8 DAMs in the BL-treated group (8 upregulated), 27 DAMs in the BL+Pyr-treated group (21 upregulated and 6 downregulated), and 21 DAMs in the Pyr-treated group (16 upregulated and 5 downregulated).

Based on the hierarchical cluster analysis of DAMs belonging to sugars and the synthetic precursors for sugars, the accumulation of these compounds exhibited a significantly increasing trend in the BL+Pyr group compared to the other three groups (untreated, BL and Pyr) (**Figure 8B**). Sucrose was highly accumulated in the BL+Pyr group (2.4-fold), followed by the Pyr group (1.9-fold), and the BL group, which showed no significant increase, compared with the untreated group (**Figure S9**). In addition, the highest accumulation of other sugars, including maltose, trehalose, lactose, arabitol, isomaltulose, melibiose, turanose and cellobiose, was also achieved by the BL+Pyr treatment (**Figure 8B**).

**Figure 9:**
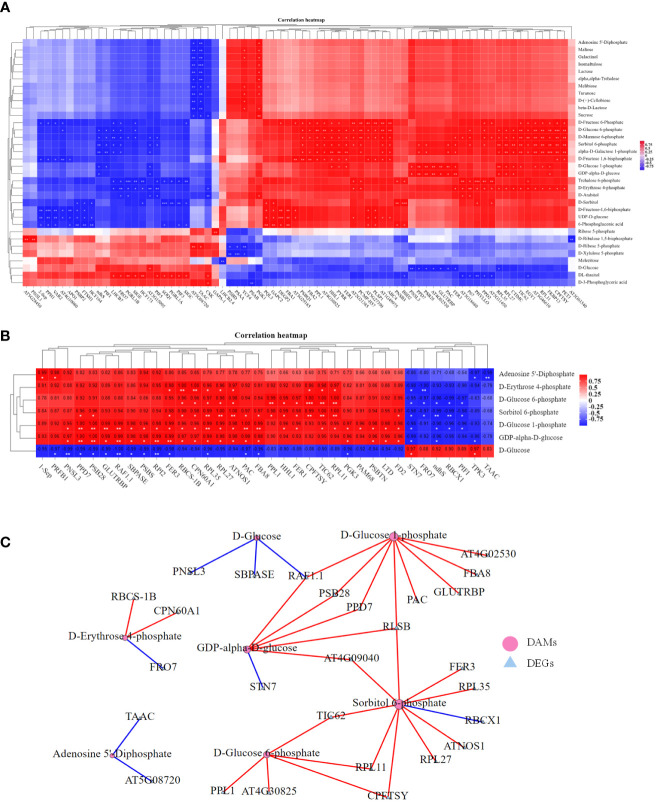
Correlation analysis of the transcriptome and metabolome profiles related to photosynthesis. **(A)** Pearson correlation analysis between photosynthesis-related DAMs and DEGs of the three treatments (BL + Pyr, BL and Pyr) versus the untreated group with correlation coefficient >0.99 and p-value<0.01. **(B)** Pearson correlation analysis between the unique photosynthesis-related DEGs and unique photosynthesis-related DAMs of the BL+Pyr group with correlation coefficient >0.95 and p-value<0.05. **(C)** The correlation network plot displaying the highly significant correlation pairs between the unique photosynthesis-related DEGs and unique photosynthesis-related DAMs of the BL+Pyr group with Pearson’s correlation coefficient >0.99 and p-value<0.01. Metabolite and transcriptome data were log2-transformed prior to correlation analysis. “*” represented 0.01< p-value<0.05, “**” represented 0.001< p-value<0.01 and “***” represented p-value ≤ 0.001.

Furthermore, hierarchical clustering analysis of DAMs in the CO_2_ fixation pathway showed that the accumulation of regenerative precursors of RuBP, including Xu5P (xylulose 5-phosphate) and R5P (ribose 5-phosphate), exhibited a decreasing trend in the BL+Pyr and Pyr groups (**Figure 8C**). Similarly, the accumulation of 3-PGA (3-phosphoglyceric acid), a key intermediate in the Calvin cycle for initiating sugar synthesis, also showed a decreasing trend in the BL+Pyr and Pyr groups (**Figure 9C**).

The contents of several photosynthates were only significantly increased in the BL+Pyr treatment group, rather than the BL or Pyr treatment groups, compared with the untreated group. The majority of these compounds are synthetic precursors of sugars, including D-glucose 6-phosphate (G1P), D-glucose 1-phosphate (G6P), GDP-D-glucose (GDPG), sorbitol 6-phosphate, galactose 1-phosphate and erythrose-4-phosphate (E4P) (**Figure 8B**). Additionally, only BL+Pyr treatment significantly increased the contents of ADP (adenosine 5’-diphosphate), which is the precursor for ATP biosynthesis *via* photosynthetic oxidative phosphorylation (**Figure 8C**).

Pearson correlation analysis of the content of the photosynthesis-related DAMs further indicated that ADP, E4P, G1P, G6P, GDPG, sorbitol 6-phosphate and galactose 1-phosphate were all positively correlated with sugars, with correlation coefficients ranging from 0.96 to 1, from 0.78 to 0.90, from 0.66 to 0.84, from 0.57 to 0.75, from 0.72 to 0.88, from 0.63 to 0.81, and from 0.56 to 0.76, respectively (**Figure S10**). Among these sugars, sucrose was the most highly correlated with these synthesis precursors of sugars, and the correlation coefficients between sucrose and ADP, E4P, G1P, G6P, GDPG, sorbitol 6-phosphate and galactose 1-phosphate were 0.99, 0.90, 0.84, 0.75, 0.88, 0.81 and 0.76, respectively (**Figure S10**). In addition, the contents of 3-PGA, Xu5P and R5P were all significantly positively correlated with the contents of those sugars, with correlation coefficients ranging from 0.92 to 0.98, from 0.83 to 0.95, and from 0.94 to 0.99, respectively (**Figure S10**).

### 3.6 Integration analysis of the transcriptome and metabolome profiles related to photosynthesis

To explore the association between the differences in the accumulation of photosynthates and the differences in the transcriptional regulation of photosynthesis among the four groups, correlation analysis between the expression level of photosynthesis-related DEGs and the accumulation of photosynthesis-related DAMs in the four groups was performed. The results showed that 160 of 243 photosynthesis-related DEGs were significantly correlated with photosynthesis-related metabolites, with a correlation coefficient >0.95 and p-value<0.05. A total of 650 significantly related pairs were identified, including 423 positively correlated pairs and 227 negatively correlated pairs (**Figure S11**). In addition, 90 of 243 photosynthesis-related DEGs and 150 significantly related pairs (90 positively correlated and 60 negatively correlated) met the correlation thresholds of Pearson’s correlation coefficient >0.99 and p-value<0.01 (**Figure 9A**). Furthermore, more DEGs were significantly correlated with the synthetic precursors of sugars than with the sugars (**Figure S11**, **Figure 9A**).

To clarify the relevance of the unique transcriptional regulation of photosynthesis to sugar accumulation in the BL+Pyr group, further correlation analysis was performed on the photosynthesis-related genes and photosynthates that were differentially regulated by the BL+Pyr treatment rather than the BL or Pyr treatments. As shown in **Figure 9B**, 36 of 79 unique photosynthesis-related DEGs in the BL+Pyr group were significantly correlated with all 7 unique photosynthesis-related DAMs in the BL+Pyr group. A total of 121 significantly related pairs were identified, including 86 positively correlated pairs and 35 negatively correlated pairs with correlation coefficients >0.95 and p-values<0.05 (**Figure 9B**). In addition, 27 of 79 photosynthesis-related DEGs and 37 significant related pairs (29 positively correlated and 8 negatively correlated) met the correlation thresholds of Pearson’s correlation coefficient >0.99 and p-value<0.01 (**Figure 9C**). Additionally, the 36 DEGs significantly associated with unique photosynthesis-related DAMs in the BL+Pyr group were involved in multiple levels of photosynthesis, such as *1-Sep, PPL1, PSBTN, PSBS, PNSL3, PPD7, PSB28, PAM68* and *STN7*, which are involved in the light reaction; *FD2, GLUTRBP, PIF1* and *NDHS*, which are involved in photosynthetic electron transport; *RBCS-1B, ATNOS1, RAF1.1, CPN60A1* and *RBCX1*, which are associated with Rubisco; and *PGK3, FBA8, SBPASE* and *RPI2*, which are associated with key enzymes in the Calvin cycle (**Figure 9B**).

To identify the photosynthesis-related core genes and metabolites specifically induced by BL+Pyr treatment, 37 highly significant correlation pairs with Pearson’s correlation coefficient >0.99 and p-value<0.01 were further selected for correlation network analysis. As shown in **Figure 9C**, more DAMs were significantly associated with *AT4G09040, CPFTSY, PPD7, PSB28, RAF1.1, RLSB* and *TIC62* (**Figure 9C**). More DEGs were significantly associated with sorbitol 6-phosphate, D-glucose 1-phosphate, GDP-alpha-D-glucose and D-glucose 6-phosphate (**Figure 9C**), indicating that these DEGs and DAMs might be core genes and metabolites for BL+Pyr treatment in synergistically improving biomass and yield.

## 4 Discussion

In this study, we comprehensively compared the effects of BL, Pyr and BL + Pyr on the biomass, yield and photosynthesis *of A. thaliana*. Exogenous application of BRs or Pyr has been reported to improve plant growth in some cases (Amaro et al., 2019; Lin, 2020; Padhiary et al., 2020). However, the growth-promoting effects of BRs are not always apparent, and Pyr inevitably causes phytotoxicity (Khripach et al., 2000; Nason et al., 2007; Vriet et al., 2012; Debona et al., 2016; Pedersen et al., 2017). Similar to previous studies, BL did not influence leaf growth when the applied dosage did not exceed 1 μM, while BL at concentrations up to 10 μM impaired leaf growth due to a disruption in the balance of the plant hormone network (**Figures S1A, B**). Additionally, Pyr affected leaf growth in a concentration-dependent manner, promoting plant growth at low concentrations and inhibiting plant growth at high concentrations due to its phytotoxicity (**Figures S1C, D**). According to **Tables S2, S3**, treatment with BL (1 μM) + Pyr (3 μM) showed the most striking leaf growth-promoting activity among all treatments, so BL (1 μM), Pyr (3 μM) and BL (1 μM) + Pyr (3 μM) were chosen for the following test to further verify the synergistic regulatory effects of BL + Pyr on promoting leaf growth. Although applying Pyr in combination with BRs has been observed to benefit plant growth in several field trials (Li et al., 2018; Li et al., 2020; Zhang, 2020; Li et al., 2021; Zhao et al., 2021), this was the first study to validate the striking synergistic effect of BL + Pyr on enhancing plant growth by precluding the benefits of disease control from Pyr.

Although BL and Pyr did not improve leaf growth during the vegetative growth period, and BL even inhibited leaf growth due to earlier bud emergence during the floral transition period (**Table 1** and **Figure 1**), BL or Pyr alone affected the growth of leaves and inflorescences in opposite ways during the reproductive development stage (**Table 2**, **3** and **Figure 2**).Pyr treatment increased the fresh weight of leaves, while BL treatment increased the number of rosette branches and effective branches and thus the fresh weight of inflorescences during the reproductive stage (**Tables 2**, **3** and **Figure 2**). Similarly, the floral transition process was delayed by Pyr and accelerated by BL according to the budding time and the flowering time (**Figures 1D**, **2A** and **Table 3**). These results suggested that Pyr benefited plants primarily by promoting the growth of vegetative organs and prolonging the vegetative growth phase, while BL benefited plants primarily by promoting the growth of reproductive organs and prolonging the reproductive growth phase. These results were consistent with previous research, as BL is extensively involved in regulating the reproductive development of plants and Pyr improves plant vegetative growth (Amaro et al., 2019; Zu et al., 2019; Li and He, 2020). Furthermore, the process of leaf senescence was delayed by Pyr, as shown by the strikingly increased fresh weight of leaves in the Pyr group at the late stage of reproductive development (**Table 2** and **Figure 2E**). This result was also consistent with the previously reported green effect of Pyr in delaying senescence (Jabs et al., 2002; Ruske et al., 2003). Yield measurements showed that yield was significantly increased by BL rather than by Pyr (**Table 3**). This result was also consistent with previous reports that Pyr benefited plants mainly by increasing biomass rather than yield in soybean (Swoboda and Pedersen, 2009), while exogenous BR could increase seed production (Tong and Chu, 2018).

Surprisingly, the yield increases achieved by BL + Pyr were almost double the total yield gain of BL or Pyr treatment alone with no accompanying variation in thousand grain weight (**Table 3**). Similarly, BL + Pyr outperformed Pyr in increasing the fresh weight of leaves in the vegetative and reproductive growth stages, apart from late reproductive development (**Tables 1**, **2** and **Figures 1**, **2**). BL + Pyr also outperformed BL in increasing the number of rosette branches and effective branches and thus the fresh weight of inflorescences (**Table 3** and **Figure 2**). Furthermore, BL + Pyr delayed the floral transition process and senescence, and these processes were delayed compared with the untreated group but accelerated compared with the Pyr group (**Table 3** and **Figure 1**, **2**). All these results suggested that the combined usage of BL + Pyr not only integrated the beneficial effects of Pyr on leaves with the beneficial effects of BL on inflorescences but also exerted a synergistic effect in enhancing biomass and yield. The increased biomass and prolonged duration of photosynthetic activity by Pyr benefited the prestorage of nutrients and BL regulated harvested organ development to reinforce the partitioning of nutrients from source to sink, which might contribute to the synergistic yield increase of BL + Pyr (Patrick and Colyvas, 2014; Mathan et al., 2016).

Improving biomass production and yield by enhancing photosynthetic capacity has been widely reported (Parry et al., 2011; Faralli and Lawson, 2020). In this study, the P_n_ was increased by the BL + Pyr treatment rather than by the BL or Pyr treatment, suggesting that the synergistic yield increase achieved by BL + Pyr was associated with improved photosynthetic capacity (**Figures 3A-B**). The corresponding increase in G_s_, T_r_, and AMC, together with deceased L_s_, in the BL + Pyr group rather than in the BL or Pyr group revealed that the enhanced photosynthesis efficiency of the BL + Pyr group was related to enhanced gas and water exchange between the photosynthetic apparatus interior and the external environment (**Figure 3**) (Kimura et al., 2020).

Furthermore, the enhanced gas exchange process in the BL + Pyr treatment did not result in a corresponding increase in intercellular CO_2_ concentration (C_i_), implying that the increased P_n_ in the BL + Pyr group might also be associated with improved CO_2_ assimilation efficiency (**Figure 3E**) (Araus et al., 2021). This assumption was further validated by the increased V_cmax_, J_max_, and V_TPU_ in the BL + Pyr group rather than in the BL or Pyr group according to CO_2_ response curves (**Figure 4**). These results demonstrated that BL + Pyr treatment enhanced the CO_2_ assimilation efficiency by increasing carboxylation efficiency, the effect of electron transport on driving the regeneration of RuBP, and triose-phosphate utilization efficiency, which contributed to the unique increase in photosynthetic efficiency induced by BL + Pyr treatment (Simkin et al., 2015; Raines, 2022).

Rubisco is the rate-limiting enzyme in the CO_2_ assimilation process and thus largely determines photosynthesis efficiency (Parry et al., 2013). Abundant studies have reported successful improvement in plant productivity and yield through overproducing Rubisco (Salesse-Smith et al., 2018; Suganami et al., 2021). So, the increased enzyme activity of Rubisco in the BL + Pyr group compared with the BL or Pyr group further supported the benefits of the BL + Pyr treatment in enhancing CO_2_ assimilation efficiency and thereby increasing the biomass and yield by enhancing photosynthetic efficiency, which were not achieved by the BL or Pyr treatments (**Figure 4B**). Meanwhile, the increased apparent quantum efficiency (AQE) by the BL+Pyr treatment rather than by the BL or Pyr treatments (**Figure S3**), further suggested that the unique benefits of the BL + Pyr treatment for photosynthesis might be related to the reduced photorespiration from increased CO_2_ concentration at the Rubisco active site(Sekhar et al., 2014). While, the corresponding increased dark respiration rate (Rd) in the BL+Pyr group might be associated with the increased availability of carbohydrates (**Figure S3** and **Figure 8B**), just as previous reported in tomato (Li et al., 2013). Additionally, this result also indicated that the increased net photosynthetic rate by the BL+Pyr treatment was not dependent on the reduction in photosynthates consumption during dark respiration.

Furthermore, compare to the untreated group, the BL+Pyr treatment, rather than the BL or Pyr treatments, improved the F_v_’/F_m_’, ΦPSII and ETR, which are positively correlated with the light harvesting and energy transduction (**Figure S4**) (Sreeharsha et al., 2015). This result supported the unique advantage of BL+Pyr treatment over the individual BL or Pyr treatments on enhancing the energy capture and utilization efficiencies by PSII and amplifying the photosynthetic electron transport efficiency. This might contribute to the production of reducing power (ATP and NADPH) for the carbon fixation process and result in the increased photosynthetic efficiency in the BL+Pyr group (Baker, 2008).

Chlorophyll a and chlorophyll b are the prime photosynthetic pigments, and they are crucial for harvesting light energy by photosynthetic antenna systems and for charge separation and electron transport within light reaction centers (Simkin et al., 2022). Many studies have proven that photosynthetic efficiency is positively correlated with chlorophyll content (Mu and Chen, 2021). However, in this study, the BL + Pyr treatment was consistent with the Pyr treatment in improving the chlorophyll a and chlorophyll b contents by upregulating genes associated with chlorophyll biosynthesis and downregulating genes associated with chlorophyll degradation (**Figure 5**). These results were not quite consistent with the overwhelming advantage of the BL + Pyr treatment over the Pyr treatment in enhancing photosynthesis, implying that the improvement in photosynthetic efficiency achieved by the BL + Pyr treatment was only partially dependent on the increase in photosynthetic pigment content.

Although exogenous BRs have been reported to positively regulate photosynthesis and alleviate the photosynthetic inhibition of plants growing under stress (Xia et al., 2009; Jiang et al., 2012; Siddiqui et al., 2018), there were no observable benefits of BL alone to the photosynthetic phenotype in this study. This contradictory result might be due to the differences in species, time of observation, application concentration, *etc.* (Zu et al., 2019; Lin, 2020; Hwang et al., 2021). In contrast, Pyr has been reported to block electron transport in photosynthesis by binding the Q_i_ site of the chloroplast cytochrome *bf* complex, thereby exerting a negative effect on photosynthesis in plants (Nason et al., 2007; Debona et al., 2016; Amaro et al., 2019). A reasonable speculation might be that Pyr only slightly inhibited photosynthesis in the plants, which was not sufficient to cause a change in the photosynthetic phenotype. However, this slight inhibitory effect of Pyr stimulated the regulatory effect of BL on photosynthesis to a greater extent, resulting in a striking increase in photosynthetic efficiency in the BL+Pyr group.

The expression profile of photosynthesis-related genes based on transcriptome analysis suggested a more significant activation of photosynthetic gene transcription by the BL+Pyr treatment than by the BL or Pyr treatment according to the following evidence. First, the BL+Pyr group had far more upregulated genes than downregulated genes compared with the other 3 groups (BL, Pyr and untreated) (**Figure 6**). Similarly, the majority of photosynthesis-related DEGs that were differentially regulated in the BL+Pyr treatment group rather than in the BL and Pyr treatment groups were also upregulated (**Figure S7**). Second, Pyr treatment and BL treatment upregulated the transcript levels of genes in the photosynthetic light reaction pathway and carbon fixation pathway, respectively (**Figure S6**). The BL+Pyr treatment not only integrated the advantages of the BL and Pyr alone in upregulating the transcription of genes in the photosynthetic pathway and the carbon fixation pathway but also was more effective than either BL or Pyr alone in upregulating the transcription of genes related to both the photosynthetic and carbon fixation pathways (**Figure 7**). Third, the GO and KEGG enrichment analysis of the photosynthesis-related genes that were differentially regulated by the BL+Pyr treatment, rather than by the BL and Pyr treatments, further indicated that BL+Pyr treatment resulted in unique transcriptional activation at multiple levels of photosynthesis, particularly in photosystem assembly, electron transport and CO_2_ assimilation (**Figure S8**).

It has been proven that photosynthetic performance can be improved by enhancing the expression of key genes involved in photosynthesis to increase the yield potential of diverse crops (Simkin et al., 2015; Simkin et al., 2019; Theeuwen et al., 2022). Therefore, the BL+Pyr treatment increases photosynthesis, and thus, the effects on biomass and yield might stem from its transcriptional activation of photosynthesis (**Figure 6**, **S7**). Photosynthesis comprises a light reaction and carbon reduction reaction (Mu and Chen, 2021). Light reactions are catalyzed by four major protein complexes, namely, PSI, PSII, cytochrome *b6f* complex (*Cyt b_6_f*), and adenosine triphosphate (ATP) synthase, which provide the reducing power (ATP and NADPH) for the carbon fixation process (Stirbet et al., 2020). The carbon fixation pathway is responsible for carbon assimilation by a series of enzymatic reactions, which are responsible for converting light energy to biomass and yield (Michelet et al., 2013). Thus, the synergistic effect of the BL+Pyr treatment on increasing photosynthesis might be associated with its transcriptional activation of the light and dark reaction of photosynthesis (**Figure 7**, **Figure S8**).

The positive contribution of improved photosynthetic efficiency to yield is achieved through the increased production of sugars, as carbohydrates derived from sugars account for more than 90% of plant biomass, making them a key determinant of yield (Ruan, 2014; Nuccio et al., 2015). Based on the metabolomic analysis, the BL+Pyr treatment was better than the BL or Pyr treatment in promoting the accumulation of sugar metabolites, including sucrose (**Figure 8** and **S9**). This result bridged the link between the synergistic enhancement of photosynthetic efficiency and synergistic increase in yield with improved sugar accumulation in the BL+Pyr group. The decreased accumulation of intermediates in the CBC cycle did not contradict the increased sugar content after BL+Pyr treatment, as the contents of 3-PGA, Xu5P and R5P were all significantly positively correlated with the contents of those sugars (**Figure 8C** and **S10**). This result consistent with previous reports that the appropriately reduced contents of CBC cycle intermediates were beneficial for improving carbon fixation efficiency and sugar synthesis (Sharkey et al., 1986; Borghi et al., 2019; Stitt et al., 2021).

The integration analysis of the transcriptome and metabolome profiles related to photosynthesis suggested that the transcription levels of photosynthesis-related DEGs were highly correlated with the accumulation of carbohydrates (**Figure S11** and **Figure 9A**). Additionally, according to the correlation analysis of the photosynthesis-related genes and the photosynthates that were differentially regulated by the BL+Pyr treatment rather than the BL or Pyr treatment, the advantage of BL+Pyr treatment in promoting photosynthate accumulation was highly correlated with the unique trans-regulation of photosynthesis at multiple levels (**Figures 9B-C**). Based on correlation analysis of sugar metabolite content, the unique advantage of the BL+Pyr treatment in enhancing sugar accumulation, particularly sucrose accumulation, might be related to the higher levels of sugar and ATP synthesis precursor accumulation than those observed in the other three groups (**Figure S10**). Furthermore, more DEGs were significantly correlated with synthetic precursors of sugars than with sugars in the integration analysis of the photosynthesis-related transcriptome and metabolome profiles (**Figure S11** and **Figure 9A**). These results implied that BL+Pyr mainly increased the production of the synthetic precursors of sugars through transcriptional regulation of photosynthesis, leading to improved accumulation of sugars.

Among the core DEGs that participate in the synergistic increase in biomass and yield after the BL+ Pyr treatment, most DAMs were associated with *RLSB* and *RAF1.1*, which are important photosynthetic regulatory proteins that regulate the translation and assembly of Rubisco, respectively (**Figure 9C**) (Yerramsetty et al., 2016; Xia et al., 2020). This result was consistent with the increased rubisco enzyme activity that was uniquely observed in the BL+ Pyr group, implying that the core genes and metabolites identified by the integration analysis of the photosynthesis-related transcriptome and metabolome profiles might contribute to exploring candidate target genes and compounds for increasing yields (**Figure 9C**).

## 5 Conclusion

**Figure 10:**
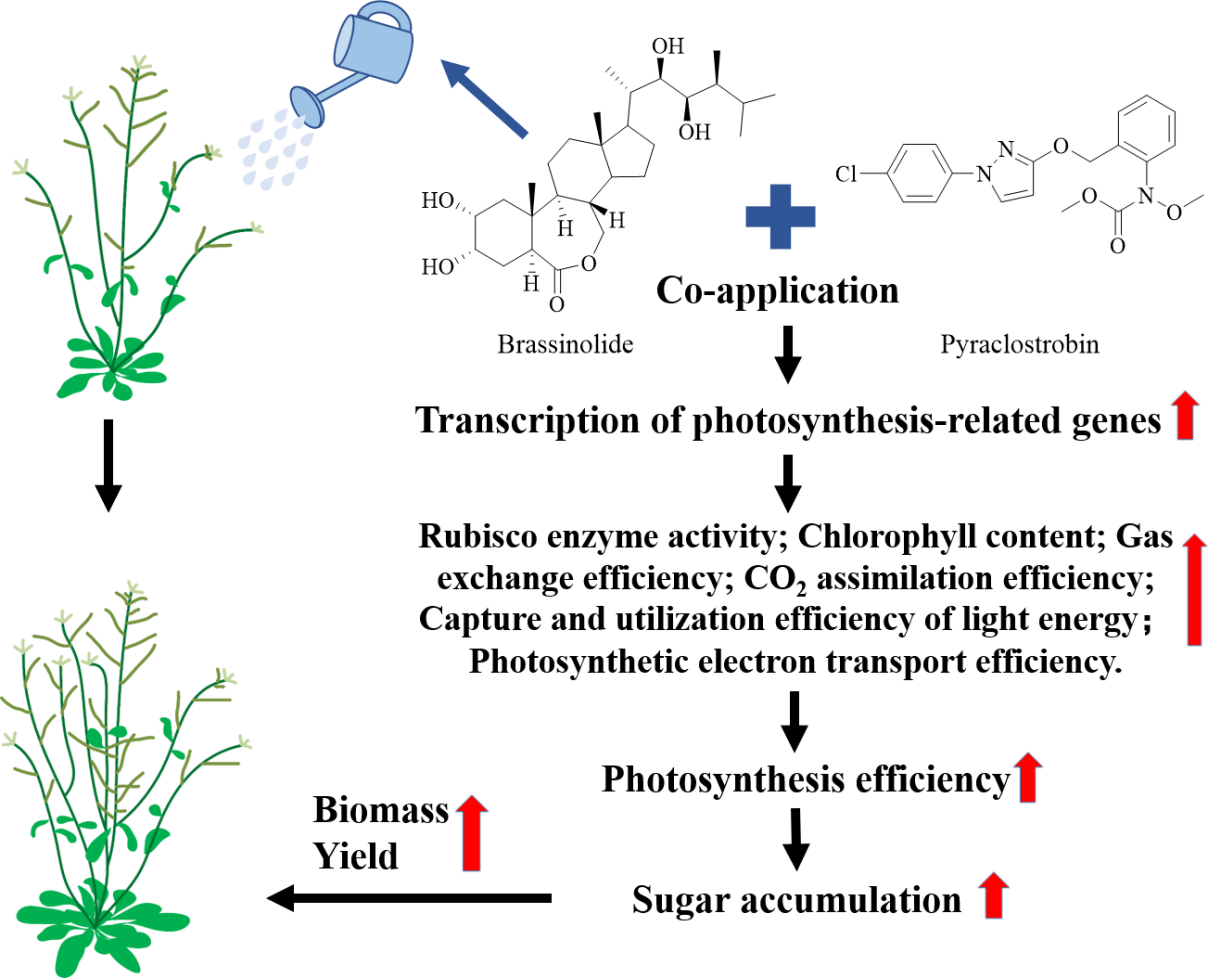
A potential model for the combined application of BL+Pyr in synergistically increasing biomass and yield through improving photosynthetic efficiency.

In summary, we first demonstrated the synergistic effect of the BL + Pyr treatment on increasing the biomass and yield by a range of phenotypic analyses throughout the full growth cycle in *A. thaliana*, and this effect occurs independently of the intrinsic fungicidal effect of Pyr and outperformed the additive effect of individual BL or Pyr treatments. The potential mechanism underlying this synergistic enhancement effect of the coapplication of BL + Pyr was shown to be closely associated with the increased photosynthesis efficiency (**Figure 10**). The BL + Pyr treatment showed an overwhelming advantage over the individual BL or Pyr treatments in enhancing the gas exchange process, CO_2_ assimilation efficiency, capture and utilization efficiency of light energy, and photosynthetic electron transport efficiency. Additionally, both BL + Pyr and Pyr treatments improved the chlorophyll a and chlorophyll b contents by upregulating genes related to chlorophyll biosynthesis and downregulating genes related to chlorophyll degradation. Based on the transcriptomic analysis, the potential mechanism by which the BL+Pyr treatment regulates photosynthesis was further revealed, and it was related to the upregulation of the transcription levels of key genes involved in multiple levels of photosynthesis. Furthermore, the BL+Pyr treatment was superior to the individual BL or Pyr treatments in increasing sugar accumulation, according to the metabolomic analysis. This result formed a bridge between the synergistic increase in photosynthetic efficiency and the synergistic increase in yield achieved by the BL+Pyr treatment. The integrated analysis of photosynthesis-related DEGs and DAMs validated the correlation between transcriptional regulation of photosynthesis at multiple levels and increased accumulation of sugars achieved by the BL+Pyr treatment. All these results potentially revealed the synergistic mechanisms by which the BL + Pyr treatment boosted the photosynthetic efficiency by increasing the transcripts of photosynthesis-related key genes, which resulted in increased carbohydrate accumulation and thus improved biomass and yield. Despite the ecotoxicology of Pyr, the revelation of the synergistic action and the potential mechanisms of the BL+Pyr treatment verified that simultaneous application of specific compounds with different targets could generate a synergistic effect in increasing plant productivity. This further provided a rational guideline for designing new eco-friendly productivity-enhancing agents. Furthermore, the identification of photosynthesis-related core genes and metabolites uniquely induced by the BL+Pyr treatment might contribute to exploring potential candidate genes and compounds for increasing yield through regulating photosynthesis.

## Data availability statement

The data presented in the study are deposited in into the NCBI’s SRA database with the link of https://www.ncbi.nlm.nih.gov/sra/PRJNA930055, under the accession number SAMN32982539 to SAMN32982550.

## Author contributions

Y-QA and ZX contributed to the conception and design of the study. Y-QA and Z-TQ performed the experiments and collected the data. D-DL, R-QZ and B-SB organized the database and performed the statistical analysis. Y-QA prepared the manuscript. D-JM, D-WW and ZX contribute to revising the manuscript. All authors contributed to the article and approved the submitted version.
