# Supplementary material for: The combined formulation of brassinolide and pyraclostrobin increases biomass and seed yield by improving photosynthetic capacity in Arabidopsis thaliana
Source: Front Plant Sci. 2023 Mar 29;14:1138563. doi: 10.3389/fpls.2023.1138563 (PMC10090558; doi:10.3389/fpls.2023.1138563)
Supplement: Supplementary file 1 [file DataSheet_1.pdf]

Supplementary Material

The combined formulation of brassinolide and pyraclostrobin increases biomass and seed yield by improving photosynthetic capacity in *Arabidopsis thaliana*

Ya-Qi An*, Zi-Ting Qin, Dan-Dan Li, Rui-Qi Zhao, Bo-Shi Bi, Da-Wei Wang, De-Jun Ma, and Zhen Xi*

*** Correspondence:**

Ya-Qi An
112018043@mail.nankai.edu.cn
Zhen Xi
zhenxi@nankai.edu.cn

1 Supplementary Figures and Tables

1.1 Supplementary Figures

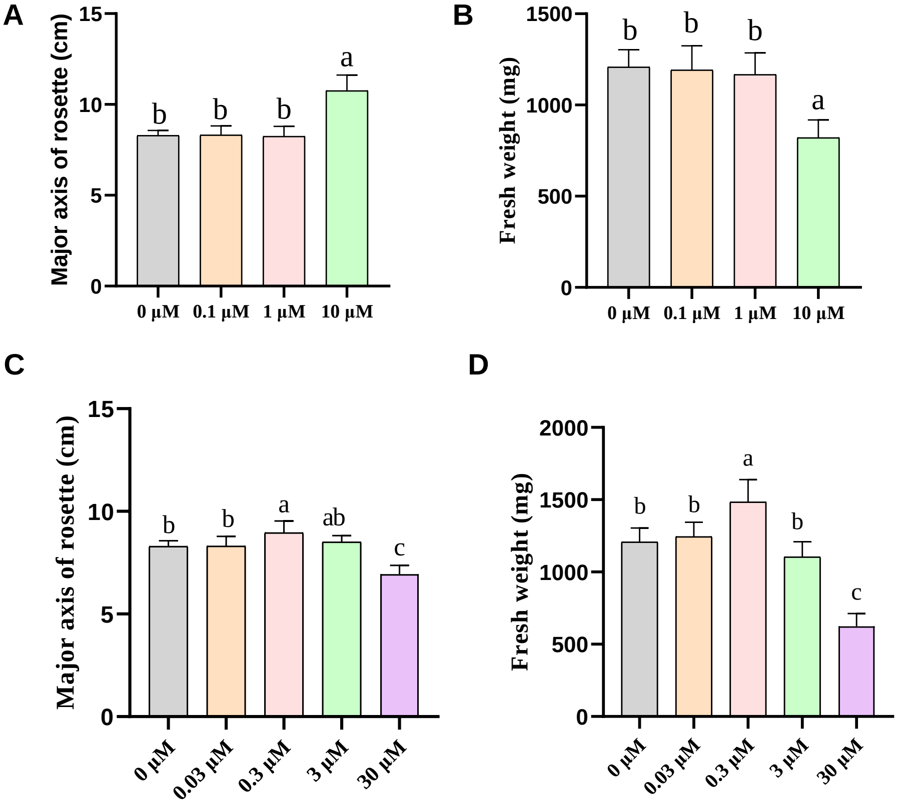

Figure S1: Effect on leaf growth with different concentrations of BL and pyraclostrobin. The fresh weight of rosette (A) and major axis (B) of BL concentrations 0.1, 1, 10 μM applied groups; The major axis (C) and fresh weight of rosette (D) of pyraclostrobin (Pyr) concentrations 0, 0.3, 3, 30 μM applied groups. Data was measured on the 15th day after application. Data was presented as the mean ± SD of three separate replicate experiments. Different letters indicated significant differences ($p < 0.05$) according to ANOVA followed by Tukey's test.

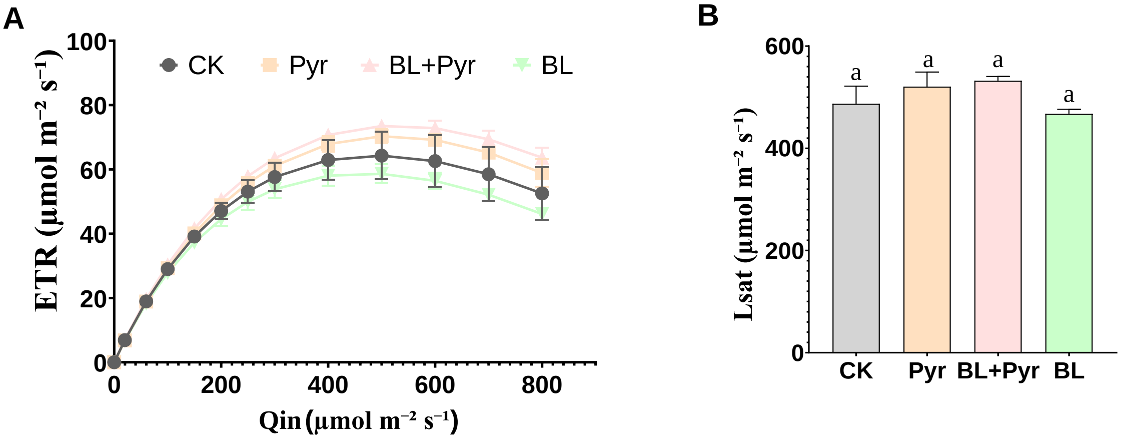

Figure S2: Rapid J-light response curve of 31-day old seedings at CK (untreated group), Pyr (3μM pyraclostrobin treated group), BL+Pyr (1μM BL and 3μM pyraclostrobin co-treated group), and BL (1μM BL treated group). A: The electron transfer rate (ETR) at different light intensity (800, 700, 600, 500, 400, 300, 250, 200, 150, 100, 60, 20, 0 μmol m^{-2} s^{-1}); B: The saturated light intensity (L_{sat}, μmol m^{-2} s^{-1}); Data are presented as the mean ± SD and each data point was the mean of three independent experiments. Different letters indicate significant differences ($p < 0.05$) according to ANOVA followed by Tukey's test.

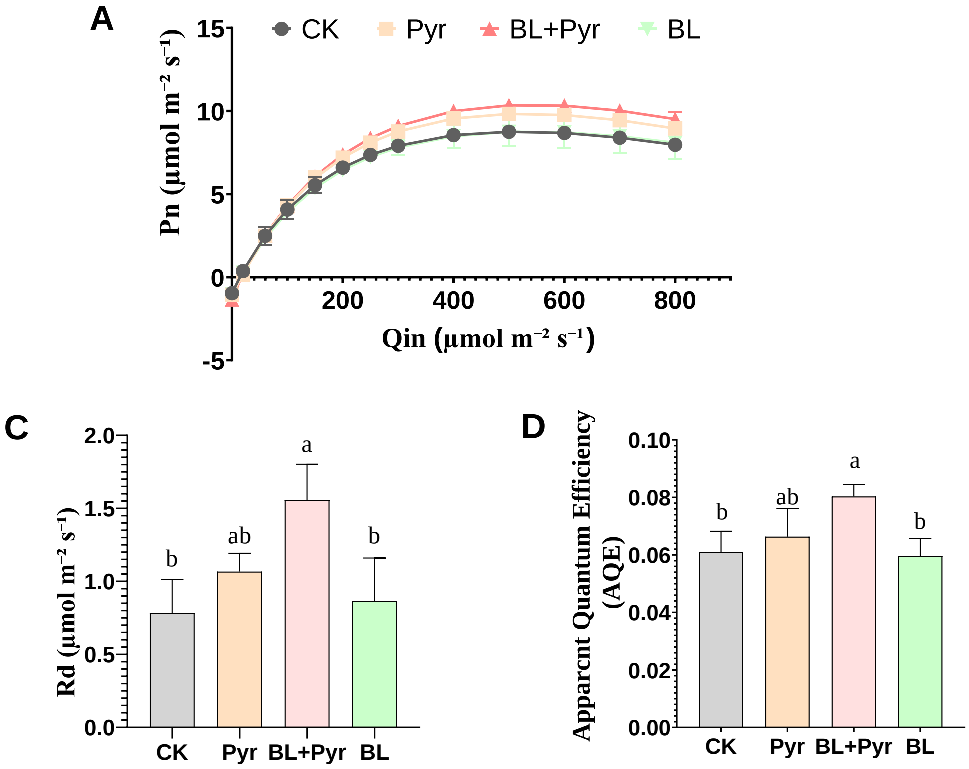

Figure S3: Rapid A-light response curve of 31-day old seedings at CK (untreated group), Pyr (3μM pyraclostrobin treated group), BL+Pyr (1μM BL and 3μM pyraclostrobin co-treated group), and BL (1μM BL treated group). A: The net photosynthetic rate (P_n) at different light intensity (800, 700, 600, 500, 400, 300, 250, 200, 150, 100, 60, 20, 0 μmol m^{-2} s^{-1}); B: The dark respiration rate (Rd, μmol m^{-2} s^{-1}); C: The apparent quantum efficiency (AQE). Data are presented as the mean ± SD and each data point was the mean of three independent experiments. Different letters indicate significant differences ($p < 0.05$) according to ANOVA followed by Tukey's test.

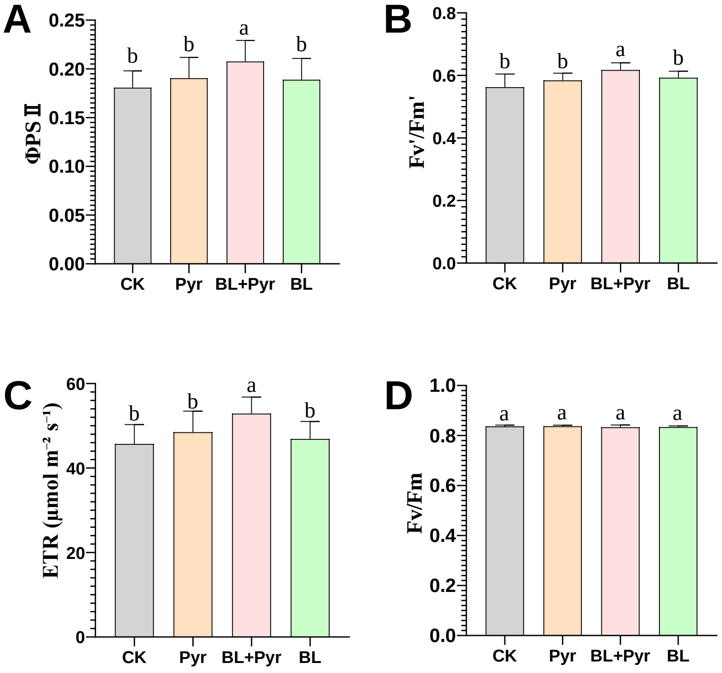

Figure S4. BL+Pyr showed a synergistic effect on increasing the energy captured and utilization efficiencies of photosynthesis. A: Quantum yield of PSII (ΦPSII); B: Efficiency of energy capture by open PSII (Fv'/Fm'); C: Electron transfer rate (ETR); D: Maximum quantum yield of PSII (Fv/Fm). Data are presented as the mean ± SD of three independent replicate experiments. Different letters indicate significant differences ($p < 0.05$) according to ANOVA followed by Tukey's test. 31-day-old seedlings (the 11th day after the first-round application) were used. CK: untreated seedlings; Pyr: seedlings treated with 3 μM pyraclostrobin; BL+Pyr: seedlings treated with 1 μM BL and 3 μM pyraclostrobin; BL: seedlings treated with 1 μM BL.

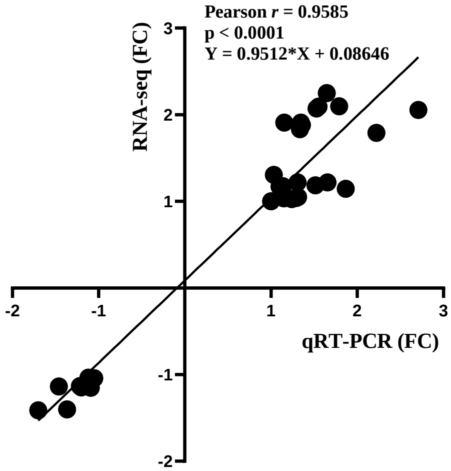

Figure S5. qRT-PCR confirmation of RNA-seq data. Ten photosynthesis-related DEGs of the BL+Pyr, BL, and Pyr groups versus the untreated group were analyzed by qRT-PCR. Correlation analysis between fold change (FC) data from RNA-seq (y-axis) and qRT-PCR (x-axis) for ten

selected genes (*PSAB, PSAA, PSAF, PSBA, ATPD, CPN60A1, RBCL, RCA, SBPASE, CFBP*) was carried out. Each data point was the mean of three independent experiments.

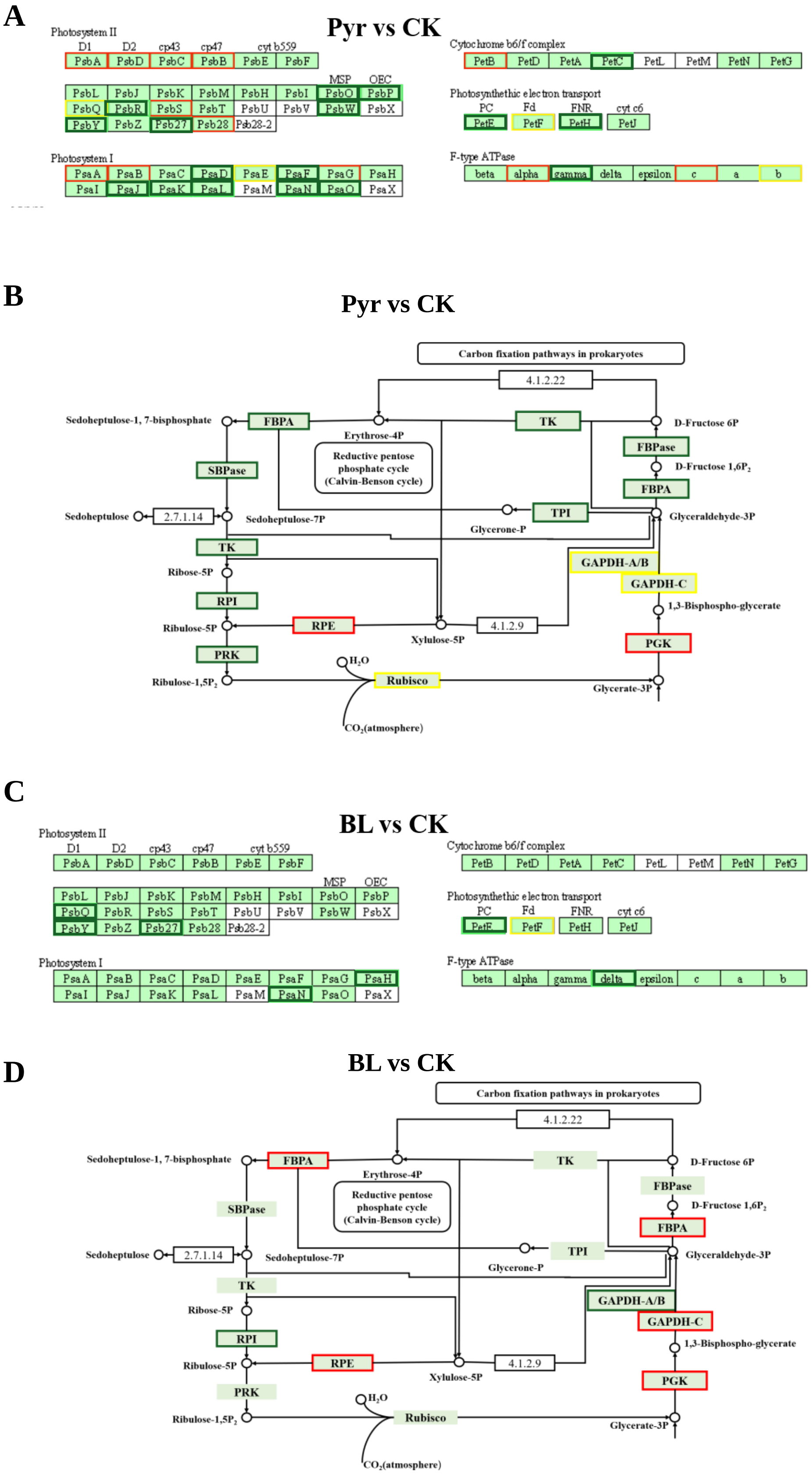

Figure S6. BL or Pyr treatments showed the opposite effect on transcription of genes in photosynthesis and carbon fixation pathway. A: Photosynthesis pathway tagged with DEGs of Pyr-treated group versus untreated group; B: Carbon fixation pathway tagged with DEGs of Pyr-treated group versus untreated group; C: Photosynthesis pathway tagged with DEGs of BL-treated group versus untreated group; B: Carbon fixation pathway tagged with DEGs of BL-treated group versus untreated group. The different colored boxes on the protein names indicate that the gene encoding the protein is either up- or down-regulated by BL+Pyr-treated group versus untreated group. CK: untreated; Pyr: Treated with 3 μM pyraclostrobin; BL: Treated with 1 μM BL. Abbreviations: SBPase: Sedoheptulose-1,7-bisphosphatase, FBPA: Fructose-1,6-bisphosphate aldolase, FBPase: Fructose-1,6-bisphosphatases, TK: Transketolase, RPE: Ribulose-phosphate 3-epimerase, TPI: Triosephosphate isomerase, GAPDH: Glyceraldehyde 3-phosphate dehydrogenase, PGK: Phosphoglycerate kinase, RPI: Ribose 5-phosphate isomerase A, PRK: Phosphoribulokinase.

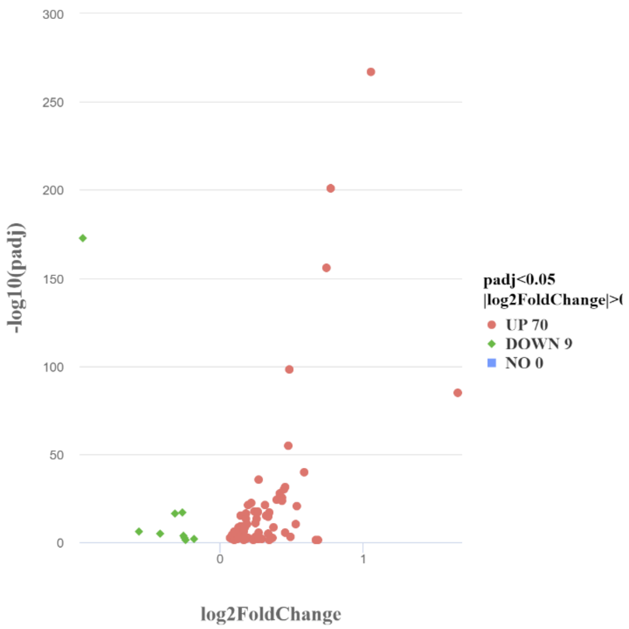

Figure S7. The volcano map of the differently expressed photosynthesis-related genes in the BL+Pyr group, but not in the BL or Pyr groups, when compared with the untreated group.

A

	ID	Descrption
**Biological processes**	GO:0015979	photosynthesis
	GO:0006091	generation of precursor metabolites and energy
	GO:0019684	photosynthesis, light reaction
	GO:0015977	carbon fixation
	GO:0019253	reductive pentose-phosphate cycle
	GO:0019685	photosynthesis, dark reaction
	GO:0006094	gluconeogenesis
	GO:0019319	hexose biosynthetic process
	GO:0006006	glucose metabolic process
	GO:0009767	photosynthetic electron transport chain
**Cellular components**	GO:0009507	chloroplast
	GO:0044434	chloroplast part
	GO:0009579	thylakoid
	GO:0009534	chloroplast thylakoid
	GO:0034357	photosynthetic membrane
	GO:0009535	chloroplast thylakoid membrane
	GO:0009570	chloroplast stroma
	GO:0009941	chloroplast envelope
	GO:0009521	photosystem
	GO:0009523	photosystem II

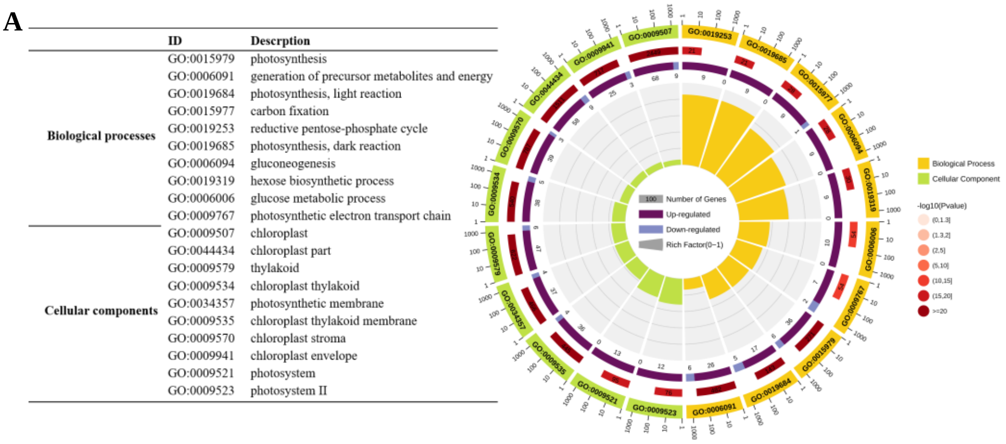

B

class	ID	Name
Metabolism	ko00710	Carbon fixation in photosynthetic organisms
Metabolism	ko00195	Photosynthesis
Metabolism	ko01200	Carbon metabolism
Metabolism	ko00010	Glycolysis / Gluconeogenesis
Metabolism	ko00030	Pentose phosphate pathway
Metabolism	ko01230	Biosynthesis of amino acids
Metabolism	ko00051	Fructose and mannose metabolism
Metabolism	ko00196	Photosynthesis - antenna proteins
Metabolism	ko01110	Biosynthesis of secondary metabolites

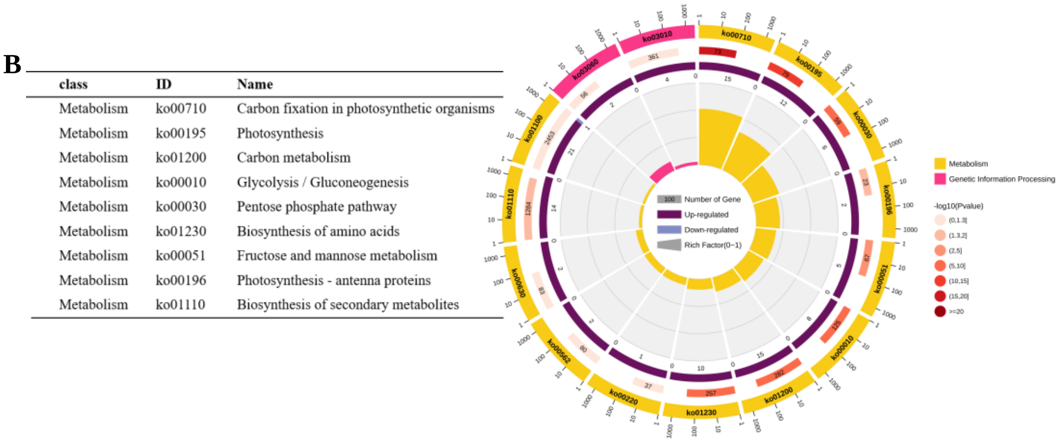

Figure S8. BL+Pyr treatment uniquely activated the transcript abundance of multiple processes in photosynthesis. A: Enrichment circular plot for top 20 significantly enriched GO terms of photosynthesis-related DEGs in BL+Pyr-treated group versus untreated group; B: Enrichment circular plot for top 15 significantly enriched KEGG pathway of photosynthesis-related DEGs in BL+Pyr-treated group versus untreated group.

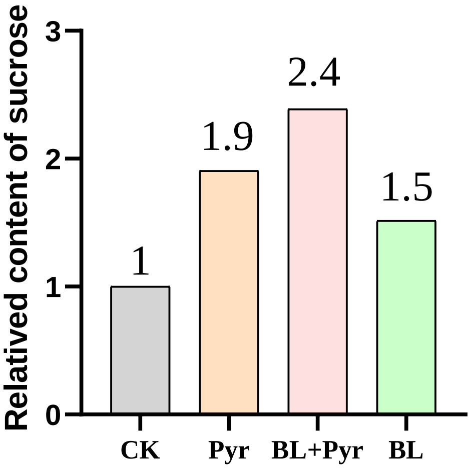

Figure S9. The average fold of sucrose content relative to the CK (untreated) group at the Pyr group (treated with 3 μM pyraclostrobin), BL+Pyr group (co-treated with 1 μM BL and 3 μM pyraclostrobin), and BL group (treated with 1 μM BL).

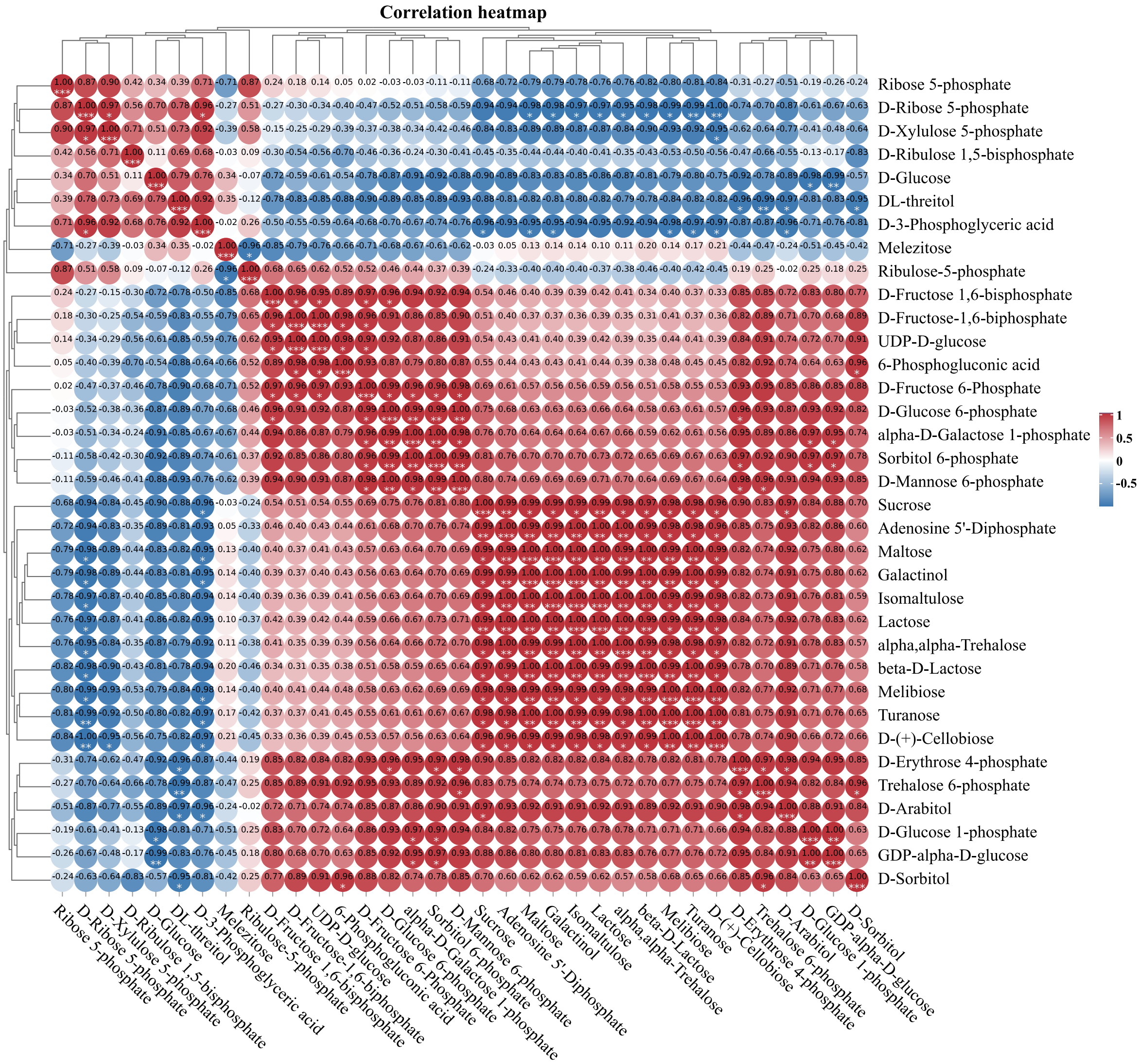

Figure S10. Pearson correlation analysis of differential accumulated photosynthates in the three groups (BL + Pyr, BL and Pyr) versus the untreated group. The values in the boxes represent Pearson correlation coefficients.

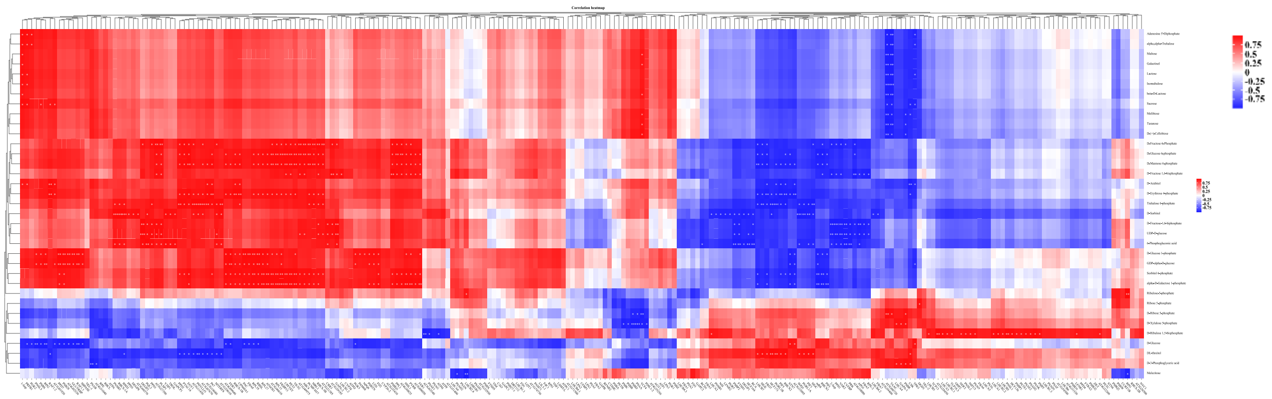

Figure S11. Pearson correlation analysis between the expression level of photosynthesis-related DEGs and the accumulation of photosynthesis-related DAMs in the four groups. Only significantly related pairs with correlation coefficient >0.95 and p-value <0.05 were shown. “*” represented 0.01< p-value <0.05, “**” represented 0.001< p-value <0.01 and “***” represented p-value≤0.001.

1.2 Supplementary Tables

gene	primer sequence
SBPASE-qRT-F	TGAGTTCTTGCTTCTTGATG
SBPASE-qRT-R	CTTGCTGTATTCGGAGTTG
RBCL-qRT-F	TACTGGTACATGGACAACTG
RBCL-qRT-R	AGTAACCGAACCTTCTTCAA
PSBA-qRT-F	GCTCCTCCAGTAGATATTGAT
PSBA-qRT-R	CCGTTGTATAGCCATTCATC
CFBP-qRT-F	CTTACTCCGCAAGGTACATT
CFBP-qRT-R	ACACTCATACAACAGCCTAAG
CPN60A-qRT-F	TCCGTCCTCTGTTCTTCC
CPN60A-qRT-R	CGTCTGATTGTCCTCTTGT
PSAB-qRT-F	TTACCTGCTTATGCGTTCAT
PSAB-qRT-R	CGTTATCCTCATTCTGTTCTG
ATPD-qRT-F	GATCAATATCGTGACGGAGA
ATPD-qRT-R	CAAGACTCGCATCAATAACC
YCF-qRT-F	CAGTCCATCAGAATAGAAGT
YCF-qRT-R	CGCAAGAAGTAAGCCAAT
PSAF-qRT-F	TCTTGCTCTCAATGCTCAG
PSAF-qRT-R	TGGTCTCCGTTCACTATCA
PSAA-qRT-F	TGTGACGGTATTGATACTGT
PSAA-qRT-R	CATCCAGAATAGTCCTAAGAAG
ACTIN2-qRT-F	TGTGCCAATCTACGAGGGTTT
ACTIN2-qRT-R	TTTCCCGCTCTGCTGTTGT

Table S1. The prime list for qRT-PCR.

BL (μM) →/ Pyr (μM) ↓	0	0.1	1	10
0	8.29±0.28b(B)	8.32±0.50b(B)	8.24±0.54b(C)	10.75±0.87a(A)
0.03	8.35±0.48b(B)	8.43±0.40b(B)	8.47±0.69b(B)	11.06±0.77a(A)
0.3	8.87±0.52b(A)	9.29±0.66b(A)	9.6±0.49ab(B)	10.17±0.52a(A)
3	8.51±0.31b(AB)	8.41±0.53b(AB)	**10.82±0.77a(A)**	10.01±0.58a(A)
30	6.84±0.56 a (C)	7.37±0.46a(C)	7.61±0.63a(C)	7.08±0.46a(B)

Table S2. The major axis (cm) of rosette leaves at CK (untreated group), Pyr (0.03, 0.3, 3, 30 μM pyraclostrobin treated group), BL+Pyr (BL 0.1, 1, 10 μM co-treated with pyraclostrobin 0.03, 0.3, 3, 30 μM, separately), and BL (0.1, 1, 10 μM BL treated group). Data was measured on the 15th day after application (45-day-old seedlings). Data was presented as the mean ± SD of three separate replicate experiments. Different lowercase letters in the same row and different capital letters in the same column both indicate significant differences ($p < 0.05$) according to ANOVA followed by Tukey's test.

BL (μM) →/ Pyr (μM) ↓	0	0.1	1	10
0	1208±95a(B)	1244±131a(B)	1231±144a(C)	776±96b(A)
0.03	1244±99a(B)	1157±178a(B)	1220±189a(C)	794±135b(A)
0.3	1564±146a(A)	1513±130a(A)	1500±104a(B)	780±115b(A)
3	1183±134b(B)	1274±132b(B)	1754±123 a(A)	731±62c(A)
30	549±83c(C)	863±73b(C)	1054±84a(C)	728±99b(A)

Table S3. The fresh weight(mg) at CK (untreated group), Pyr (0.03, 0.3, 3, 30 μM pyraclostrobin treated group), BL+Pyr (BL 0.1, 1, 10 μM co-treated with pyraclostrobin 0.03, 0.3, 3, 30 μM, separately), and BL (0.1, 1, 10 μM BL treated group). Data was measured on the 15th day after application (45-day-old seedlings). Data was presented as the mean ± SD of three separate replicate experiments. Different lowercase letters in the same row and different capital letters in the same column both indicate significant differences ($p < 0.05$) according to ANOVA followed by Tukey's test.

It should note that the Table S4 and Table S5 are demonstrated in Excel file named Table S4 and Table S5.
